# Phytochemical profiling, antiviral activities, molecular docking, and dynamic simulations of selected *Ruellia* species extracts

**DOI:** 10.1038/s41598-024-65387-5

**Published:** 2024-07-04

**Authors:** Mina Michael Melk, Ahmed F. El-Sayed

**Affiliations:** 1 Pharmacognosy Department, Faculty of Pharmacy, Ahram Canadian University, Giza, Egypt; 2 Microbial Genetics Department, Biotechnology Research Institute, National Research Centre, Giza, Egypt; 3 Egypt Center for Research and Regenerative Medicine (ECRRM), Cairo, Egypt

**Keywords:** Phytochemical profiling, Antiviral, *Ruellia*, Docking, Molecular dynamics, Antivirals, Plant sciences, Analytical chemistry, Virtual drug screening

## Abstract

The antiviral properties of the flowering aerial extracts of *Ruellia tuberosa* and *Ruellia patula* were investigated through phytochemical profiling via LC–MS/MS and HPLC techniques. Qualitative LC–MS/MS analyses identified seventy-seven metabolites from both *Ruellia* species. *R. tuberosa* had the highest phenolic content (49.3%), whereas *R. patula* had the highest flavonoid content (57.8%). Additionally, quantitative HPLC investigations of the compounds identified by LC–MS/MS were performed using the available standard compounds. The main constituents in the *R. tuberosa* extract was found to be catechin (5321.63 µg/g), gallic acid (2878.71 µg/g), and ellagic acid (2530.79 µg/g), whereas the major compounds in the *R. patula* extract was found to be rutin (11,074.19 µg/g) and chlorogenic acid (3157.35 µg/g). Furthermore, the antiviral activities of both *Ruellia* species against HAdV-40, herpes simplex type 2 and H1N1 were evaluated. These findings demonstrated that *R. tuberosa* was more active than *R. patula* against all tested viruses, except for the HSV-2 virus, against which *R. patula* showed greater activity than *R. tuberosa*, with IC_50_ values of 20, 65, 22.59, and 13.13 µg/ml for *R. tuberosa* flowering aerial parts and 32.26, 11.66, and 23.03 µg/ml for *R. patula* flowering aerial parts, respectively for HAdV-40, herpes simplex type 2, and H1N1. Additionally, computational docking and molecular dynamics simulations were used to assess the molecular interactions between the bioactive compounds and specific viral targets. The combined findings from the in-vitro and in-silico experiments comprehensively evaluated the antiviral activities of both *Ruellia* species extracts.

## Introduction

Infections caused by numerous viral strains in humans are accountable for millions of deaths worldwide. These include illnesses like hepatitis, influenza, herpes simplex, HIV/AIDS, and the common cold^1^. There are more than 70 genotypes of nonenveloped double-stranded DNA viruses known as adenoviruses (ADVs). Human beings recognize seven species of ADVs (A–G). Human ADVs are generally not highly pathogenic and are primarily linked to gastroenteritis, hemorrhagic cystitis, and self-limiting respiratory infections, especially in newborns and early childhood^2^. Human adenovirus can impact the gastrointestinal system, respiratory tract, and ocular surface, among other human organs. Adults with compromised immune systems and children are particularly vulnerable to adenoviral infections^3^. Ocular ADV infection is one of the main causes of viral conjunctivitis in humans. Human ADV infections lead to significant morbidity and mortality in immunocompromised patients, such as those receiving hematopoietic stem cell transplantation or solid organ transplants^4^. According to reports, human ADV is the most prevalent virus among transplant patients, particularly in pediatric units^5^. Since there are currently no FDA-approved antivirals for this type of virus, alternative anti-adenoviral therapies must be developed^6^.

The influenza A (H1N1) virus, first identified in North America in March 2009, spread rapidly worldwide through human-to-human contact, posing a major public health threat^7^. Two types of antiviral medications—neuraminidase inhibitors and M2 ion channel inhibitors—have been authorized for the treatment and prevention of influenza. However, the effectiveness of these FDA-approved antiviral medications has been limited by the emergence of drug-resistant strains, rapid development of resistance during treatment, and potential central nervous system side effects^8,9^. This rise in drug-resistant influenza viruses highlights the urgent need for novel antiviral treatments. Herpes simplex virus (HSV) is a member of the herpesvirus subfamily and is a frequent pathogenic virus in humans. HSV-1 and HSV-2, also known as herpes simplex viruses, are linear double-stranded DNA viruses with complex spherical structures composed of at least 18 proteins and a four-layer structure^10,11^. Herpes simplex virus keratitis, conjunctivitis, cold sores, and other illnesses can be caused by HSV-1. Blindness is usually caused by HSV-1-induced recurrent eye infections^12^. Commonly generating infections of the central nervous system, HSV-1 can cause severe focal necrotizing encephalitis with a high clinical death rate and a dismal prognosis, leaving patients with several neurological abnormalities^13^. Currently, valaciclovir (VCV) and acyclovir (ACV), two nucleoside analogs, are often utilized in clinical treatment^14^. However, these medications are ineffective in the early stages of infection and neither eradicate the virus from the host nor stop HSV from recurring^15^. Antiviral medications are widely used, which can cause the virus to mutate and become more resistant to HSV-1^16–18^. Thus, it’s becoming increasingly important to investigate novel medications, such as alternative nonnucleoside analogs and organic substances, that are effective against HSV-1.

The lack of effective treatments for viral illnesses significantly burdens public healthcare systems. Therefore, the development of affordable, low-toxicity, and broad-spectrum antiviral medications is a major goal for both pharmaceutical research and virology^19^. However, current antiviral medications often come with serious side effects like kidney damage or addiction, potentially hindering future treatment options^1^. This highlights the urgent need for more potent antivirals with fewer side effects. Herbal antiviral medications have gained increasing attention in recent decades due to their broad-spectrum antiviral properties. Natural products have been shown to potentially reduce side effects, lower toxicity, alleviate symptoms of some viral infections, and shorten their duration^20^. This has led to the development of various drug delivery technologies, such as solid dispersions, micelles, nanoparticles, and self-emulsifying systems, that efficiently and reliably deliver these natural antiviral compounds.

There are approximately 150 species in the *Ruellia* genus (Acanthaceae), which is also referred to as *Dipteracanthus*. Most of them are bushes and herbs that can be found in tropical and subtropical areas, including Malaysia, Africa, Pakistan, Brazil, Indonesia, and Central America. This genus has historically been associated with the treatment of rheumatic complaints, eczema, fever, flu, asthma, hypertension, bronchitis, diabetes, paronychia, venereal illnesses, sores, tumors, and antimicrobial and hepatoprotective activity conditions^21–24^. The chemical constituents of the genus *Ruellia* include flavonoids, which are the predominant constituents within this genus, such as cirsimaritin, cirsimarin, cirsiliol 4′-glucoside, sorbifolin, luteolin 7-*O*-glucoside, apigenin, apigenin 7-*O*-glucoside, apigenin 7-*O*-glucuronide, quercetin, quercetin 3-*O*-glucoside, demethoxycentaureidin, hispidulin, comanthoside B, pectolinaringenin, nepetin 7-*O*-β-d-glucopyranoside, and pectolinarigenin; lignans such as rupaside, lyoniresinol glycoside, and syringaresinol glycoside; coumarins such as 7-hydroxy-4-methyl coumarin and dicoumarol; alkaloids such as tetramethylputrescine and indole-3-carboxaldehyde; triterpenes such as lupeol, betulin, and *β*-amyrin; sterols such as *β*-sitosterol, *β*-sitosterol glucoside, stigmasterol, and campesterol; phenolics such as vanillic acid, *p*-methoxy benzoic acid, *p*-coumaric acid, vanilloside, and syringin; and phenyl ethanoids such as acteoside, isoacteoside, nuomioside, isonuomioside, forsythoside B, paucifloside, cassifolioside, isocassifolioside, cistanoside E, and castanoside^25^.

According to previous phytochemical studies of the genus *Ruellia*, especially *R. tuberosa* Linn. and *R. patula* Jacq, flavonoid glycosides, together with lignans, phenolic glycosides, megastigmane glycosides, benzoxazinoid glucosides, and sterols, were the major constituents of the plants under investigation^25^. The various biological effects reported in the literature showed scarcity in the antiviral assessment of both plants despite the presence of active antiviral constituents in the genus *Ruellia,* such as phenolics and flavonoids^26^. This prompted us to explore the active constituents of aerial floral extracts from both *Ruellia* species and to study their antiviral effects on Adenovirus type 40 (ADV 40), influenza A (H1N1), and Herpes simplex virus type 2 (HSV-2). Furthermore, the molecular docking method was also used to mimic the atomic-level interaction between a small molecule and a protein^27^.

## Materials and methods

### Plant materials

The aerial flowering parts of *R. tuberosa* and *R. patula* were acquired from the El Orman Botanical Garden in Giza, Egypt, after authorization for purchasing the plant materials was obtained. The validity of the plants was confirmed by Mrs. Therese Labib, the El-Orman Botanical Garden's Consultant in Plant Identification. Voucher specimens 17,092,023 and 19,092,023 were obtained from the Department of Pharmacognosy, Faculty of Pharmacy, Ahram Canadian University. A total of 250 g of air-dried material was subjected to long cold maceration with 70% ethanol and frequent stirring. When separated and dried under vacuum at 40 °C, the extraction yields for *R. patula* and *R. tuberosa* were 24.57% and 23.62%, respectively.

### LC-ESI-TOF–MS analysis of the aq.-Ethanolic extract

#### Sample preparation

A 1:1:10 mixture of water, methanol, and acetonitrile (H_2_O: MeOH: ACN) was used to prepare a stock solution from 50 mg of the previously obtained aqueous-ethanolic extract from *Ruellia* species. The sample was completely dissolved using vortexing and 10 min of ultrasonication at 30 kHz. To prepare the injection solution, a small portion (20 µL) of the stock solution was diluted with 1000 µL of H_2_O: MeOH: ACN (2:1:1) and centrifuged at 10,000 rpm for 5 min. Finally, 10 µL (equivalent to 1 µg/mL) of the solution was injected for LC–MS analysis. Blank, quality control, and internal standard (IS) samples were also analyzed alongside the sample for experimental confidence. Notably, both positive and negative ionization modes were used during the LC–MS analyses^28^.

#### Instruments and acquisition method

Small molecules were separated using an ExionLC system (AB Sciex, Framingham, MA, USA) connected to an autosampler system, an XBridge C18 (3.5 µm, 2.1 × 50 mm) column (Waters Corporation, Milford, MA, USA), and an inline filter disk precolumn (0.5 µm × 3.0 mm, Phenomenex, Torrance, CA, USA). The temperature was maintained at 40 °C, and a flow rate of 300 µL/min was used. The pH of solution (A), which included 5 mM ammonium formate in 1% methanol and was adjusted to 3.0 with formic acid, and solution (B), which contained 100% acetonitrile for the positive mode, composed the mobile phase. The negative mode solution (C) was made up of 1% methanol and 5 mM ammonium formate, which was adjusted with sodium hydroxide to a pH of 8. The following sequence was used for gradient elution: 0–20 min, 10% B; 21–25 min, 90% B; 25.01–28 min, 10% B; and finally, 90% B for column equilibration. A Triple TOF 5600 + system with a Duo-Spray source running in the ESI mode (AB SCIEX, Concord, ON, Canada) was used for the mass spectrometry (MS) experiments. In the positive mode, the sprayer capillary and declustering potential voltages were 4500 V and 80 V, respectively, and in the negative mode, they were − 4500 V and − 80 V, respectively. Gas 1 and gas 2 were set at 40 psi, the curtain gas was at 25 psi, and the source temperature was fixed at 600 °C. The collision energy was set at 35 and − 35 V for the positive and negative modes, respectively with a 20 V CE spread and an ion tolerance of 10 ppm. Information-dependent acquisition (IDA) protocol was used to run the Triple TOF 5600+. Analyst-TF 1.7.1 was used to build batches for the gathering of MS and MS/MS data. Data from both full-scan MS and MS/MS were simultaneously gathered using the IDA technique. High-resolution survey spectra spanning from 50 to 1100 m/z were employed in the technique, and the mass spectrometer was set up to detect survey scans every 50 ms. Following each scan, the top 15 strong ions were chosen to obtain MS/MS fragmentation spectra^28^.

#### LC–MS data processing

The material was thoroughly examined using small molecules and non-targeting methods with the open-source MS-DIAL 3.70 program. ReSpect-positive (2737 records) or ReSpect-negative (1573 records) databases were used as reference databases based on the acquisition mode. To gather the data for peak detection, the following search parameters were used: minimum peak height, 100 amplitude; mass slice width, 0.05 Da; smoothing level, 2 scans; minimum peak width, 6 scans; and identification for alignment. The tolerances were as follows: MS1 and MS2, 0.2 Da each; retention time, 0.05 min; and MS1, 0.25 Da. For feature (peak) confirmation from the total ion chromatogram (TIC), the MS-DIAL output was used once more to run on PeakView 2.2 with the MasterView 1.1 package (AB SCIEX). The criteria used were aligned features with a signal-to-noise ratio greater than 5 and sample intensities greater than 5 (blank)^28^.

### HPLC analyses

The Agilent 1260 series was used for HPLC analysis. An Eclipse C18 column (4.6 mm × 250 mm i.d., 5 μm) was used for separation. Water (A) and 0.05% trifluoroacetic acid in acetonitrile (B) were the components of the mobile phase, which was mixed at a rate of 0.9 ml/min. The following sequential linear gradient program was used for the mobile phase: 8–12 min (60% A), 0–5 min (80% A), 12–15 min (82% A), 15–16 min (82% A), and 16–20 min (82% A). At 280 nm, a multiwavelength detector was used. For every sample solution, there was one injection volume of five microliters. At 40 °C, the column temperature was kept constant^29–31^.

### Antiviral assessment

#### Assessment of the antiviral activity against human adenovirus type 40 (HAdV-40)

The assessment was conducted using Hep-2 cells and human adenovirus type 40. Hep-2 cells were cultured in DMEM supplemented with 0.1% antibiotic/antimycotic solution and 10% fetal bovine serum. Fetal bovine serum, trypsin–EDTA, DMEM, and antibiotic and antimycotic solutions were obtained from Gibco BRL (Grand Island, NY, USA). Using the recently described cytopathic (CPE) inhibitory effect, antiviral activity and cytotoxicity were assessed using the crystal violet method. To summarize, one day before infection, hep-2 cells were plated at a density of 2 × 104 cells/well in a 96-well culture plate. The following day, the culture media was removed, and phosphate-buffered saline was used to wash the cells. The crystal violet method was utilized to determine the infectivity of human adenovirus type 40. This method allowed for calculating the percentage of cell viability while also monitoring CPE. Mammalian cells were exposed to 0.1 ml of a diluted human adenovirus type 40 viral suspension comprising CCID50 (1.0 × 10^4^) of the virus stock. Three to four days after infection, this dosage was chosen to result in the expected CPEs. The cells were treated with compounds by adding 0.01 ml of media with the required extract concentration. The antiviral activity of each test sample was assessed using a concentration range of 0.1–1000 μg/ml that had been diluted twice. Both the cell controls (noninfected, nondrug-treated cells) and the viral controls (virus-infected, nontreated cells) were included. The culture plates were cultured in 5% CO_2_ at 37 °C for 96 h. The progression of cytopathic effects was observed via light microscopy. After being washed with PBS, the cell monolayers were fixed and stained with a 0.03% crystal violet solution in 2% ethanol and 10% formalin. Following cleaning and drying, the optical density of each well was measured using a spectrophotometer set at 570/630 nm. Pauwels et al. provided the following equation, which was used to determine the percentage of antiviral activities of the test compounds: antiviral activity = [(mean optical density of cell controls − mean optical density of virus controls)/(optical density of test − mean optical density of virus controls)] × 100%. The 50% CPE inhibitory dose (IC_50_) was computed in light of these findings^32^. We evaluated the cytotoxicity before this experiment by seeding cells in a 96-well culture plate at a density of 2 × 104 cells/well. The following day, the cells were exposed to culture media containing serially diluted samples. The medium was removed after 72 h, and the cells were washed with PBS. The subsequent procedures were performed identically to those previously described for the antiviral activity test. GraphPad PRISM software (GraphPad Software, San Diego, USA) was used to calculate the 50% inhibitory concentration (IC_50_) and 50% cytotoxic concentration (CC_50_)^33^.

#### Assessment of the antiviral activity against influenza virus (H1N1)

Madin-Darby canine kidney (MDCK) cells and influenza virus (H1N1) were cultured in DMEM supplemented with 10% fetal bovine serum and 0.1% antibiotic/antimycotic solution. Fetal bovine serum, trypsin–EDTA, DMEM, and antibiotic and antimycotic solutions were obtained from Gibco BRL (Grand Island, NY, USA). The cytopathic effect (CPE), which was recently discovered, was utilized to assess antiviral activity and cytotoxicity assays utilizing the crystal violet method^34,35^. To summarize, one day before infection, MDCK cells were seeded at a density of 2 × 104 cells/well into a 96-well culture plate. The following day, the culture media was removed, and phosphate-buffered saline was used to wash the cells. The crystal violet method was used to monitor CPE, and the percentage of cell viability was calculated to estimate the infectivity of the H1N1 virus. Mammalian cells were treated with 0.1 mL of a diluted H1N1 virus suspension comprising CCID50 (1.0 × 10^6^) of the virus stock. To achieve the necessary CPEs two days postinfection, this dosage was chosen. The cells were treated with extracts by adding 0.01 ml of media with the required extract concentration. The antiviral activity of each test sample was measured at doses that were diluted two times, beginning at 1000 μg/ml. Both the cell controls (noninfected, nondrug-treated cells) and the viral controls (virus-infected, nontreated cells) were included. The culture plates were cultured in 5% CO_2_ at 37 °C for 72 h. The progression of cytopathic effects was observed via light microscopy. After being washed with PBS, the cell monolayers were fixed and stained with a 0.03% crystal violet solution in 2% ethanol and 10% formalin. Following cleaning and drying, the optical density of each well was measured using a spectrophotometer set at 570/630 nm. The following formula was used to determine the percentage of antiviral activity of the test compounds: antiviral activity × (mean optical density of cell controls − mean optical density of virus controls)/(optical density of test − mean optical density of virus controls) × 100^32^. The 50% CPE inhibitory dose (IC_50_) was computed considering these findings. We evaluated the cytotoxicity before this experiment by seeding cells in a 96-well culture plate at a density of 2 × 10^4^ cells/well. The following day, the cells were exposed to culture media containing serially diluted samples. The medium was removed after 72 h, and the cells were washed with PBS. The subsequent procedures were performed identically to those previously described for the antiviral activity test. GraphPad PRISM software (GraphPad Software, San Diego, USA) was used to calculate the 50% inhibitory concentration (IC_50_) and 50% cytotoxic concentration (CC_50_)^36^.

#### Assessment of the antiviral activity against the human herpes simplex virus type 2

Vero cells were cultured in DMEM supplemented with 0.1% antibiotic/antimycotic solution and 10% fetal bovine serum. Fetal bovine serum, trypsin–EDTA, DMEM, and antibiotic and antimycotic solutions were obtained from Gibco BRL (Grand Island, NY, USA). Using the previously discovered cytopathic (CPE) inhibitory effect, antiviral activity and cytotoxicity were assessed using the crystal violet method. To summarize, one day before infection, hep-2 cells were plated at a density of 2 × 10^5^ cells/well in a 96-well culture plate. The following day, the culture media was removed, and phosphate-buffered saline was used to wash the cells. The crystal violet method, which measures CPE and allows for calculating the percentage of cell viability, was used to assess the infectivity of HSV-2. Mammalian cells were exposed to 0.1 mL of a diluted human adenovirus type 40 viral suspension comprising CCID50 (1.0 × 10^5^) of the virus stock. Three days following infection, this dosage was chosen to result in the intended CPEs. The cells were treated with extracts by adding 0.01 ml of media with the required extract concentration. The antiviral activity of each test sample was assessed using a concentration range of 0.1–1000 μg/ml that had been diluted twice. Both the cell controls (noninfected, nondrug-treated cells) and the viral controls (virus-infected, nontreated cells) were included. The culture plates were cultured in 5% CO_2_ at 37 °C for 96 h. The progression of cytopathic effects was observed via light microscopy. After being washed with PBS, the cell monolayers were fixed and stained with a 0.03% crystal violet solution in 2% ethanol and 10% formalin. Following cleaning and drying, the optical density of each well was measured using a spectrophotometer set at 570/630 nm. Using the following formula, antiviral activity [(mean optical density of cell controls − mean optical density of virus controls)/(optical density of the test − mean optical density of virus controls)] × 100, the percentage of antiviral activities of the test compounds was determined following Pauwels et al.^32^. The 50% CPE inhibitory dose (IC_50_) was computed in light of these findings. We evaluated the cytotoxicity before this experiment by seeding cells in a 96-well culture plate at a density of 2 × 104 cells/well. The following day, the cells were exposed to culture media containing serially diluted samples. The medium was removed after 72 h, and the cells were washed with PBS. The subsequent procedures were performed identically to those previously described for the antiviral activity test. GraphPad PRISM software (GraphPad Software, San Diego, USA) was used to calculate the 50% inhibitory concentration (IC_50_) and 50% cytotoxic concentration (CC_50_)^32^.

### Computational methods

#### Molecular docking simulation

Antibacterial protein receptors were procured from the Protein Data Bank, as specified in Table 1, to examine the antibacterial properties of the promising compounds. Using PyMOL software, the crystal structures of the target receptors were preprocessed to exclude ions, water molecules, and preexisting ligands. The receptor molecule was then modified by adding hydrogen atoms using Autodock Vina^37^ and stored in pdbqt format. Additionally, Open Babel was used to reduce each compound and convert it to a mol2 format^38^. The Autodock tools were then used to convert the compounds into pdbqt format. AutoGrid software was used to create ligand-centered maps with grid sizes of 90 Å × 90 Å × 90 Å. The remaining options were all set to their default values. AutoGrid and AutoDock Vina were used to construct the grid maps^37^. Additionally, Discovery Studio 4.5 software was used to investigate the 2-D bond interactions between the compounds and target receptors. Finally, using Lipinski's Rule of Five^39^, the ADMET (absorption, distribution, metabolism, excretion, and toxicity) properties of the drugs were calculated.

**Table 1:** List of target proteins, PDB IDs, active site coordinates, native ligands, and references.

No	Protein targets	PDB ID	Resolution Å	Active site coordinates			Reference ligands	References
				X	Y	Z		
1	Human Adenovirus type 40	**4PIE**	1.94 Å	60.4	5.24	2.54	3FO	^40^
2	Human Herpes simplex virus 2	**1AT3**	2.50 Å	20.33	2.54	8.22	DFP	^41^
3	Influenza virus (H1N1)	**3B7E**	1.45 Å	5.60	35.66	-11.18	ZMR	^42^

#### Molecular dynamics (MD) simulation

Protein–ligand complex binding affinities and interactions are frequently explained by molecular dynamics (MD) simulations along certain paths. In this study, GROMACS 2018 software was used to run 50 ns MD simulations to confirm the reliability and rationale of the docking results. The CHARMM36 force field parameters were utilized to design the topology of the viral protein^43^. The Geoff server was used to generate the Mangiferin coordinate files and topology. Following coordination, ligands are subject to limitations. At 300 K and 1.0 bar at atmospheric pressure, NVT and NPT equilibrium were measured for 500 and 1000 ps, respectively. Ultimately, each system underwent 50 ns MD simulations, with coordinate trajectories being recorded every 10 ps over the whole run. The Root Mean Square Deviation (RMSD), root mean square fluctuation (RMSF), and radius of gyration (Rg) were computed following the MD simulations.

### Institutional review board statement

The methodology and experimental design were approved by the research ethics committee of Ahram Canadian University's College of Pharmacy, and the experiments were performed in compliance with their requirements. Affirmation Number: REC2024.

## Results

### LC–MS/MS

LC–MS/MS was used to profile the phenolic metabolites in the floral aerial extracts of *R. tuberosa* and *R. patula*. Seventy-seven metabolites were detected in *R. tuberosa* and *R. patula*. combined. Based on their fragmentation pattern, molecular weight, and retention times as well as a comparison with published data from the literature and an online database called Mass Bank, the compounds were identified and provisionally annotated. According to the findings, phenolics and flavonoids are thought to be the two main active constituents found in both *Ruellia* species. *R. patula* has more flavonoids than *R. tuberosa*, with percentages of 57.8% and 38.7%, respectively, whereas *R. tuberosa* has higher proportions of phenolics than *R. patula* (49.3% and 36.4%, respectively). Compared with *R. patula*, *R. tuberosa* was shown to contain greater amounts of coumarins (8.5% and 1.2%), anthocyanidin glycosides (0.8% and 0.4%), and catechins (1.7% and 0.4%*)*. Furthermore, *R. patula* was shown to contain higher levels of hydroxycinnamic acid and its glycosides (3.7% and 1.7%, respectively), as well as stilbenes and their glycosides (0.7% and 0.6%, respectively), than *R. tuberosa*. The results of the LC–MS/MS analyses are presented in Table 2 and Supplementary Fig. S1 and structures of the identified compounds are presented in Supplementary Figs. S2 and S3.

**Table 2:** Phenolic compounds identified by LC/MS–MS from the extracts of the aerial floral parts of *R. tuberosa* and *R. patula*.

#	Metabolite name	Compound type	Average Mz	MS/MS spectrum	Error (ppm)	*R. tuberosa*		*R. patula*		References
						RT	Relative intensity	RT	Relative intensity	
1	Gallic acid	Phenolics	168.0057	79.10213, 81.099602, 96.90023, 125.01076, 168.06845	− 0.6	1.087	0.238	1.049	0.049	^44^
2	Acacetin-7-*O*-rutinoside	Flavonoid glycosides	577.1602	270.05243, 285.07668, 577.17804	− 0.2	1.148	0.734	1.11	0.077	^45^
3	d-(−)-Quinic acid	Phenolics	191.0551	59.01458, 85.03008, 93.03682, 96.0232, 146.95898, 155.02953, 191.0565	− 2.6	1.158	13.464	1.12	10.817	^46^
4	Coumaric acid	Phenolics	163.0396	93.03489, 119.05796, 162.83244	0.6	1.178	21.873	1.14	0.003	^47^
5	Pyrocatechol	Catechins	109.02902	81.03486, 108.01131, 109.03085	− 0.7	1.196	0.075	1.158	0.047	^44^
6	3,4-Hydroxybenzoic acid	Phenolics	137.0239	78.96051, 91.01811, 109.02789, 137.01703	0.0	1.203	0.055	1.165	0.031	^48^
7	3′ 4′ 5 7-Tetrahydroxyflavanone	Flavanones	287.05562	107.05042, 121.02695, 149.04338, 153.05648, 287.04419	0.1	1.328	0.194	1.29	0.069	^49^
8	Vanillin	Phenolics	151.03951	108.00486, 123.04343, 136.01605, 151.08712	0.1	1.332	6.076	1.294	0.004	^44^
9	E-4,5′-dihydroxy-3-methoxy-3′-glucopyranosylstilbene	Stilbenes and their glycosides	419.39206	92.92608, 191.05038:143, 267.07486, 373.12497, 419.07794	− 0.3	1.347	0.345	1.309	0.133	^50^
10	Methyl gallate	Phenolics	183.02965	43.056871, 121.10818, 153.01887, 183.14591	− 0.3	1.349	0.451	1.311	0.038	^44^
11	Rhoifolin	Flavonoid glycosides	577.16032	147.02383, 191.05067, 235.03879, 569.08026, 577.15826	0.0	1.361	0.225	1.323	1.311	^51^
12	p-Coumaric acid	Phenolics	163.03953	76.97284, 118.96393, 119.05329, 163.03609	0.2	1.759	0.365	1.721	0.061	^45^
13	Syringic acid	Phenolics	197.0452	58.01449, 121.20085, 138.22807, 153.0154, 181.90377, 197.00147	1.0	1.809	1.333	1.771	12.033	^44^
14	7-Hydroxy-4-methyl coumarin	Coumarins	175.03953	86.97613, 131.06199, 147.31541, 175.05512	0.2	1.818	8.134	1.78	0.921	^52^
15	Ferulic acid	Phenolics	193.05015	131.02677, 147.00115, 165.05447, 193.03591	0.3	1.894	1.581	1.856	10.002	^44^
16	Kaempferitrin	Flavonoid glycosides	577.16032	255.0231, 283.05826, 285.01843, 430.0165, 431.04034, 577.06522	0.0	1.927	0.024	1.889	0.059	^44^
17	Hesperetin	Methylated flavonoids	301.07082	125.02135, 164.01121, 283.03922, 286.06418, 301.0585	0.1	1.975	0.319	1.937	0.003	^53^
18	E-3,4,5′-Trihydroxy-3′-glucopyranosylstilbene	Stilbenes and their glycosides	405.39182	159.03992, 201.06654, 241.04697, 243.08823, 405.20883	− 0.4	2.065	0.034	2.027	0.481	^54^
19	Eriodictyol-7-*O*-neohesperidoside	Flavonoid glycosides	597.50762 [M + H]^+^	443.17505, 475.20181, 487.15646, 549.5625, 597.19012	− 0.6	2.141	0.017	2.103	0.166	^45^
20	Okanin-4′-*O*-glucoside	Flavonoid glycosides	449.39153	135.01152, 136.00371, 150.99883, 151.01978, 152.00341, 447.67889, 449.12631	− 1.0	2.153	0.097	2.115	2.871	^55^
21	Chlorogenic acid	Phenolics	353.087617	179.02759, 191.30223, 207.03508, 288.0379, 353.12482	0.0	2.191	1.135	2.153	3.104	^44^
22	Isorhamnetin-3-*O*-glucoside	Flavonoid glycosides	477.10205	119.0541, 125.08433, 145.03071, 163.04073, 177.05674, 270.04955, 300.07799, 315.10205	− 0.3	2.393	0.092	2.355	2.878	^56^
23	Syringetin-3-*O*-galactoside	Flavonoid glycosides	509.40762	153.05188, 258.03955, 287.04431, 315.06021, 331.06926, 347.01862, 509.11184	− 0.7	2.645	0.011	2.607	0.117	^45^
24	Isookanin-7-glucoside	Flavonoid glycosides	449.39252	151.00465, 241.07246, 269.05017, 287.05365, 288.05855, 313.05467, 431.12473, 449.11386	1.2	2.669	0.245	2.631	0.404	^57^
25	3 5 7-trihydroxy-4′-methoxy flavone	Flavonols	299.05516	126.01813, 141.03732, 162.06374, 164.94601, 179.03548, 299.07516	− 1.5	2.680	0.241	2.642	0.058	^45^
26	Eriodictyol-7-*O*-glucoside	Flavonoid glycosides	449.11219 [M + H]^+^	135.0218, 151.00616, 287.05759, 449.11218	0.0	2.740	0.228	2.702	0.551	^45^
27	Isoquercitrin	Flavonoid glycosides	463.08851	255.13335, 271.04495, 300.00122, 301.00759, 303.86935, 428.1192, 463.14851	2.0	3.267	0.015	3.229	0.083	^58^
28	Luteolin-3′, 7-di-*O*-glucoside	Flavonoid glycosides	609.15093	285.04568, 447.09741, 609.17493	0.5	4.226	0.011	4.188	0.166	^57^
29	Gentisic acid	Phenolics	153.01904	57.03546, 65.00278, 68.99857, 83.01341, 107.01434, 109.02961, 125.02512, 153.01904	1.6	4.270	0.119	4.232	0.076	^59^
30	Hesperetin-7-*O*-neohesperidoside	Flavonoid glycosides	609.17022	301.02496, 609.20022	− 1.6	4.748	0.020	4.71	0.026	^57^
31	Rosmarinic acid	Coumarins	359.07698	123.03621, 161.04234, 197.03318, 359.08398	2.2	4.905	0.207	4.867	0.276	^57^
32	Kaempferol-3-Glucuronide	Flavonoid-3-O-glucuronides	461.07015	113.03937, 229.04799, 285.07843, 461.1301	− 2.9	4.954	0.078	4.916	0.309	^45^
33	Datiscin	Flavonoid	593.52268	125.02344, 151.02501, 165.00446, 266.90134, 285.04745, 449.11142, 593.14468	1.1	4.968	0.016	4.93	0.160	^57^
34	Cinnamic acid	Hydroxycinnamic acid and its glycosides	147.03916	100.03109, 106.08893, 131.03021	− 2.3	5.060	1.185	5.022	1.079	^44^
35	Quercetin-3-Glucuronide	Flavonoid glucuronides	477.35235	135.02425, 151.00388, 258.02451, 301.01523, 477.06735,	0.7	5.228	0.548	5.19	2.260	^60^
36	1-*O*-b-d-glucopyranosyl sinapate	Hydroxycinnamic acid and its glycosides	385.11841	190.0979, 205.0463, 385.17441	− 1.0	5.310	0.474	5.272	2.634	^57^
37	(-−)-Epicatechin	Catechins	289.07095	122.03832, 137.02351, 138.03214, 187.04137, 188.04706, 273.0694, 287.14825, 289.06955	− 0.9	5.312	0.057	5.274	0.086	^61^
38	Esculetin	Coumarins	177.01933	77.04007, 89.03796, 105.03532, 133.03034, 177.01933	3.0	5.326	0.155	5.288	0.012	^62^
39	(+)-3 3′ 4′ 5 7-Pentahydroxyflavan	Catechins	301.22196	95.04548, 111.98637, 151.00073, 188.94098, 201.91121, 229.08134, 247.9007, 285.0731, 289.073	− 0.1	5.401	0.034	5.363	0.045	^51^
40	Isorhamnetin-3-*O*-rutinoside	Flavonoid glycosides	623.16085	271.07336, 285.00302, 300.02658, 315.00656, 461.0011, 477.01984, 623.21185	0.1	5.466	0.020	5.428	0.038	^63^
41	Delphinidin-3-*O*-(6″-*O*-alpha-rhamnopyranosyl-beta-glucopyranoside)	Anthocyanidin glycosides	610.15597	125.03149, 299.0177, 300.02469, 301.05093, 423.06958, 609.12897	0.1	5.682	0.045	5.644	0.081	^64^
42	Quercetin-3-*O*-arabinoglucoside	Flavonoid glycosides	595.49066	300.02567, 557.2207, 559.21606, 595.12866	− 2.3	5.701	0.042	5.663	0.961	^65^
43	Quercetin-3,4′-*O*-di-beta-glucopyranoside	Flavonoid glycosides	625.50914	300.05283, 301.02115, 463.08167, 557.23407, 625.12659	0.2	5.891	0.062	5.853	0.314	^66^
44	Apigenin 8-C-glucoside	Flavonoid glycosides	431.09707	59.01431, 71.01865, 169.01688, 179.05983, 283.05856, 311.05484, 313.07812, 322.95779, 341.17377, 397.19421, 431.18707	− 1.2	5.903	0.098	5.865	0.161	^57^
45	Quercetin	Flavonols	301.03491	137.0217, 152.04773, 228.07591, 301.10599	0.0	6.031	0.016	5.993	0.366	^63^
46	Myricitrin	Flavonoid glycosides	463.08761	151.00418, 179.00124, 214.02783, 242.0218, 243.0291, 271.02386, 287.0188, 316.02103, 317.02856, 463.08441	0.0	6.123	0.189	6.085	0.172	^44^
47	Kaempferol-3-*O*-(6-p-coumaroyl)-glucoside	Flavonoid -O-p-coumaroyl glycosides	593.51153	1459.07101, 227.043, 255.02377, 284.03705, 285.03043, 400.98029, 447.04181, 593.14453	− 0.1	6.142	0.071	6.104	0.037	^57^
48	Cyanidin-3-*O*-(2″-*O*-beta-xylopyranosyl-beta-glucopyranoside)	Anthocyanidin glycosides	580.49354	255.0381, 284.02594, 533.0647, 541.2113:290, 543.22058, 579.13354	4.4	6.227	0.035	6.189	0.270	^68^
49	Catechin	Catechins	289.07126	82.98071, 96.9592, 112.98394, 188.9438, 244.90843, 289.11606	0.2	6.752	0.670	6.714	0.005	^44^
50	Quercetin-3-d-xyloside	Flavonoid glycosides	433.08263	243.03117, 255.02933, 271.02142, 300.02295, 301.03653, 387.19949, 433.07449	0.1	6.901	0.549	6.863	6.529	^57^
51	Kaempferol-3-*O*-glucoside	Flavonoid glycosides	447.09769	227.03845, 255.02979, 284.03107, 285.04349, 447.09406	0.2	6.919	5.731	6.881	5.809	^44^
52	Kaempferol-3-*O*-alpha-l-arabinoside	Flavonoid glycosides	417.342302	227.03387, 255.02756, 284.03143, 285.03436, 417.08444, 417.2402	0.7	7.312	1.169	7.274	0.786	^45^
53	Quercetin-7-*O*-rhamnoside	Flavonoid glycosides	447.09715	179.00002, 227.0251, 254.03107, 255.02917, 71.02103, 284.03278, 285.02853, 294.89264, 300.02704, 301.03815, 362.87607, 447.09015	− 1.0	7.324	11.617	7.286	12.850	^69^
54	Phlorizin	Flavonoid glycosides	435.12943	119.05179, 123.04491, 167.02745, 167.03474, 169.19914, 273.07675, 350.90799, 435.12943	1.0	7.435	0.029	7.397	0.072	^64^
55	Naringenin-7-*O*-glucoside	Flavonoid glycosides	433.10855	151.00311, 271.06219, 285.03754, 300.02917, 301.03296, 364.89182, 433.24081	0.8	7.585	0.274	7.547	0.296	^57^
56	Daidzein-8-C-glucoside	Isoflavonoid glycosides	415.10366	253.05005, 267.02426, 295.10482, 369.19711, 415.19366	2.3	7.641	0.110	7.603	0.898	^57^
57	Kaempferol-3-*O*-alpha-l-rhamnoside	Flavonoid glycosides	431.3711	227.03552, 229.05087, 255.02931, 284.03329, 285.04099, 431.0957	2.6	7.722	7.737	7.684	7.542	^57^
58	Peonidine-3-*O*-glucoside chloride	Anthocyanidin glycosides	497.89202	271.026, 299.01822, 300.03009, 314.04044, 315.04984, 461.10724	0.0	7.785	0.047	7.747	0.065	^68^
59	Daidzein	Isoflavonoids	253.05012	132.1088, 223.14267, 253.04712	0.1	7.986	6.273	7.948	0.010	^44^
60	Rutin	Flavonoid	609.15012	151.0191, 271.08478, 301.08343	− 0.8	8.314	0.020	8.276	0.024	^67^
61	Baicalein-7-*O*-glucuronide	Flavonoid-7-O-glucuronides	445.35489	113.9845, 175.04652, 269.04654, 298.04471, 445.10889	2.0	9.306	0.021	9.268	0.009	^71^
62	Quercetin-3-Arabinoside	Flavonoid glycosides	433.08277	255.03386, 271.02377, 299.9863, 300.02786, 301.03656, 387.21899, 433.19504	0.4	9.675	0.037	9.637	0.330	^72^
63	Resveratrol	Stilbenes and their glycosides	227.0704	114.9883, 143.04947, 158.97037, 185.05505, 227.0704	− 1.8	9.774	0.008	9.736	0.003	^44^
64	3′-Methoxy-4′,5,7-Trihydroxyflavononol	Flavonols	315.05048	246.89272, 269.16931, 287.01871, 299.99115, 300.99979, 315.01248	− 0.1	9.850	0.581	9.812	0.517	^66^
65	Quercitrin	Flavonoid glycosides	447.097615	271.03119, 300.02832, 301.03702, 401.25342, 447.09091	0.0	10.731	0.345	10.693	7.894	^57^
66	Kaempferide	Flavonol glycosides	299.05505	92.92916, 93.03402, 119.05172, 121.02762, 149.99791, 151.00452, 164.01129, 165.02206, 227.02716, 255.04372, 270.04883, 284.07513	− 1.8	13.569	0.071	13.531	0.007	^64^
67	3 3′ 4′ 5-tetrahydroxy-7-methoxy flavone	Flavonols	317.061879 [M + H]^+^	123.04649, 134.03596, 149.06369, 165.01666, 243.99927, 246.89548, 271.00251, 299.9942, 315.02313, 315.08578	− 0.1	13.809	0.021	13.771	0.006	^46^
68	Ellagic acid	Phenolics	301.18932	135.04401, 184.03796, 229.08897, 257.93954, 283.08496, 285.19641	1.1	14.748	1.486	14.71	0.183	^45^
69	Apigenin	Flavones	269.04521	66.00492, 89.01464, 117.0327, 118.05347, 151.03426, 182.07402, 269.08221,	0.8	14.796	0.018	14.758	0.003	^67^
70	Kaempferol	Flavonoid	285.04099	92.03112, 142.65563, 187.00648, 227.03552 285.04099	3.8	15.052	0.230	15.014	0.033	^73^
71	Naringenin	Flavanones	271.06089	119.02618, 151.00104, 177.07188, 186.93256, 227.09959, 245.05777, 255.06554, 271.09589	0.7	15.960	0.007	15.922	0.003	^74^
72	Acacetin	Methylated flavonoids	283.06078	211.08983, 239.04178, 268.24832	0.3	16.058	0.007	16.02	0.001	^55^
73	Luteolin	Flavones	285.03992 [M + H]^+^	133.03021, 151.01837, 166.02678, 224.07912, 267.10199, 285.11264	0.1	17.436	0.003	17.398	0.001	^67^
74	Esculin	Coumarins	339.07156	105.05778, 133.02303, 149.07631, 163.11279, 177.04336, 339.23236	1.1	22.612	0.012	22.574	0.002	^57^
75	Caffeic acid	Phenolics	179.14557	135.0662, 178.94427	− 2.4	26.970	1.093	26.932	0.008	^46^
76	Pyro catechol	Catechols	109.02912	108.96732	1.1	27.517	0.014	27.479	0.106	^44^
77	Poncirin	Flavanones	593.192631	122.96614, 285.0773, 327.0885	0.1	27.603	0.738	27.565	0.106	^64^

*RT* retention time.

### HPLC analyses

Seventeen and fourteen chemicals were found in the aerial extracts of *R. tuberosa* and *R. patula*, respectively, according to HPLC studies. Catechin (5321.63 µg/g), gallic acid (2878.71 µg/g), and ellagic acid (2530.79 µg/g) were the major compounds in *R. tuberosa* extract, while rutin (11,074.1888 µg/g) and chlorogenic acid (3157.344992 µg/g) were the major compounds in *R. patula* extract. Catechin, caffeic acid, vanillin, daidzein, and hesperetin (6673.78, 129.68, 33.70, 172.37, and 22.74 µg/g, respectively) were detected only in the *R. tuberosa* extract, while pyrocatechol and quercetin (78.85 and 267.58 µg/g, respectively) were detected only in the *R. patula* extract. This approach also revealed that the concentrations of gallic acid, ellagic acid, naringenin, and cinnamic acid were greater in *R. tuberosa* (2878.71, 2530.79, 371.47, and 66.13 µg/g, respectively), while the concentrations of chlorogenic acid, methyl gallate, syringic acid, rutin, ferulic acid, and kaempferol were greater in *R. patula* (3157.35, 359.07, 448.15, 11,074.19, 174.17, and 268.45 µg/g, respectively). Coumaric acid (80.04 and 80.36 µg/g) and apigenin (14.31 and 16.43 µg/g) were detected at comparable concentrations in both the aerial extracts of *R. tuberosa* and *R. patula*, respectively*.* The HPLC analyses are presented in Table 3 and Supplementary Fig. S4.

**Table 3:** Phenolic compounds in the aerial flowering parts of *R. tuberosa* and *R. patula were identified by HPLC*.

	*R. tuberosa*				*R. patula*			
	RT	RRT	Area^a^	Conc. (µg/g)	RT	RRT	Area^a^	Conc. (µg/g)
Gallic acid	3.388	0.39	1181.92 ± 3.26	2878.71	3.35	0.39	196.78 ±	599.90
Chlorogenic acid	4.221	0.49	240.56 ± 1.34	962.11	4.21	0.49	630.69 ± 1.05	3157.35
Catechin	4.567	0.53	999.30 ± 2.33	6673.78	–	–	–	–
Methyl gallate	5.594	0.64	139.69 ± 0.89	211.74	5.76	0.67	189.26 ± 5.03	359.074
Coffeic acid	5.919	0.68	56.77 ± 0.24	129.68	–	–	–	–
Syringic acid	6.52	0.75	38.66 ± 0.06	79.00	6.56	0.76	175.19 ± 6.02	448.15
Pyro catechol	–	–	–	–	7.05	0.82	15.66 ± 4.35	78.85
Rutin	7.856	0.90	119.78 ± 1.09	481.30	7.84	0.91	2201.79 ± 7.99	11,074.19
Ellagic acid	8.693	1.00	323.82 ± 3.48	2530.79	8.58	1.00	16.28 ± 0.71	159.25
Coumaric acid	9.104	1.05	108.98 ± 0.76	80.04	9.16	1.07	87.41 ± 2.06	80.36
Vanillin	9.815	1.13	28.88 ± 0.33	33.70	–	–	–	–
Ferulic acid	10.265	1.18	24.85 ± 1.02	46.20	10.02	1.17	74.83 ± 5.03	174.17
Naringenin	10.578	1.22	122.53 ± 3.02	371.47	10.75	1.25	87.55 ± 5.22	332.23
Daidzein	12.238	1.41	91.08 ± 4.09	172.37	–	–	–	–
Quercetin	–	–	–	–	12.60	1.47	65.37 ± 3.08	267.58
Cinnamic acid	13.916	1.60	104.60 ± 4.26	66.13	13.93	1.62	5.29 ± 0.32	4.19
Apigenin	14.479	1.67	5.29 ± 0.32	14.31	14.58	1.70	6.50 ± 0.51	16.43
Kaempferol	14.825	1.71	14.38 ± 0.77	49.05	15.09	1.76	62.85 ± 8.04	268.43
Hesperetin	15.488	1.78	14.44 ± 0.56	22.74	–	–	–	–

*RT* retention time, *RRT* relative retention time to ellagic acid. ^a^Area is expressed as the mean ± S.E.

### Antiviral activities of both *Ruellia* species

**Figure 1:**
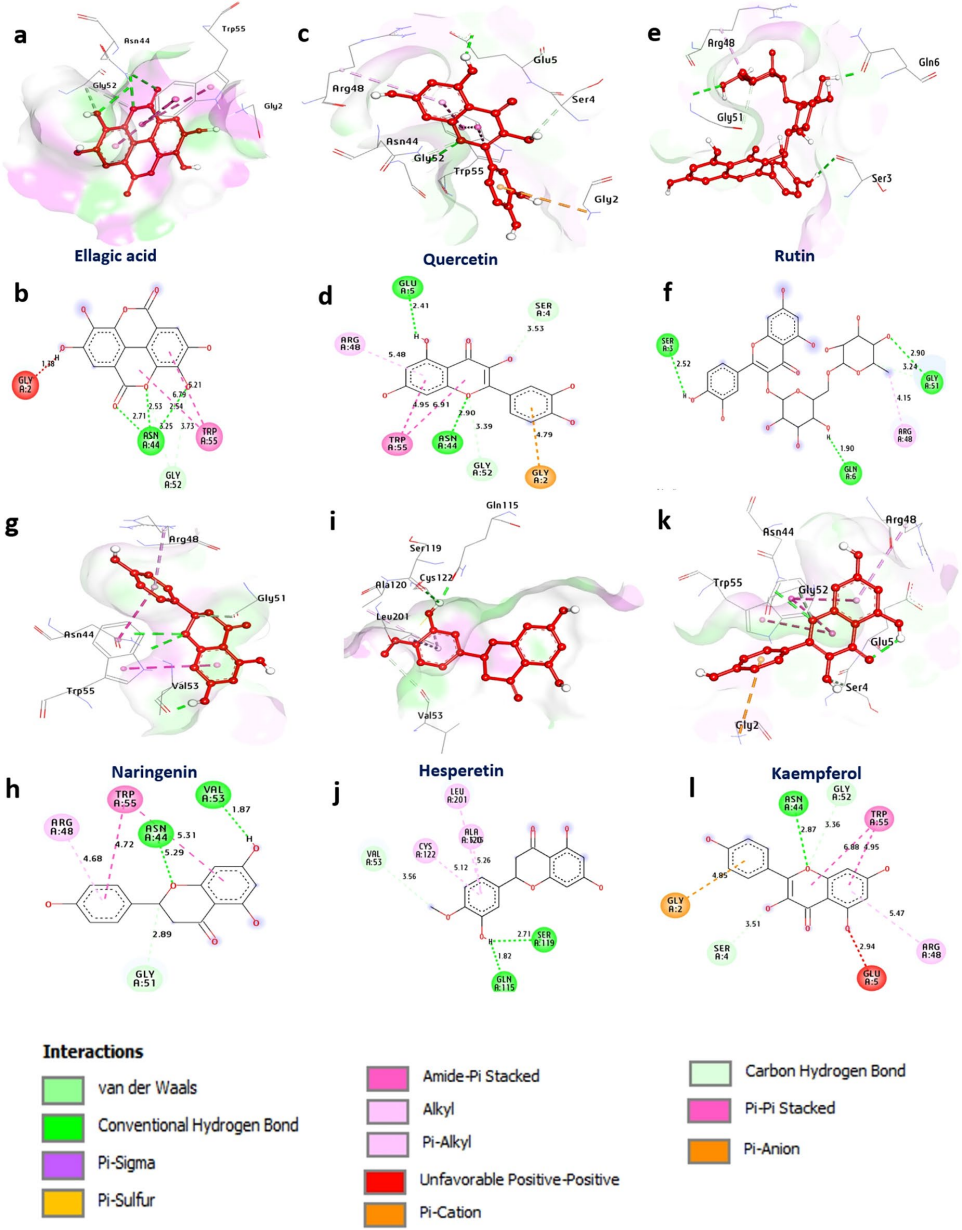
3D representations of compound conformations at the binding pocket of human adenovirus type 40 (PDB: ID 4PIE): (**a**,**b**) ellagic acid, (**c**,**d**) quercetin, (**e**,**f**) rutin, (**g**,**h**) naringenin, (**i**,**j**) hesperetin, and (**k**,**l**) kaempferol.

According to the results tabulated in Table 1 and Fig. 1, both *Ruellia* species showed antiviral activity against HAdV-40, herpes simplex type 2, and H1N1, with IC_50_ values of 20.65, 22.59, and 13.13 µg/ml, respectively, for the flowering aerial parts of *R. tuberosa* and 32.26, 11.66, and 23.03 µg/ml, respectively, for the flowering aerial parts of *R. patula*. These results showed that *R. tuberosa* is more active than *R. patula* against the tested viruses, except for the HSV-2 virus, as *R. patula* had a greater effect than *R. tuberosa*. Both *Ruellia strains* were considered safe because they showed cytotoxic effects at higher concentrations than those found for their inhibitory effects, with CC_50_ values equal to 190.188, 187.076, and 80.00 for *R. tuberosa* and 218.718, 107.913, and 129.521 for *R. patula* against the tested cell lines, Hep-2 cells, Vero cells, and Madin-Darby canine kidney (MDCK) cells for the HAdV-40, herpes simplex type 2, and H1N1 viruses, respectively. These assessments used gallic acid, acyclovir, and oseltamivir as standard drugs for HAdV-40^75^, herpes simplex^76^, and H1N1^77^., respectively. The results also showed a significant difference when compared with the values of standard drugs at P < 0.5. The results of the antiviral activities are tabulated in Table 4 and presented in Supplementary Figs. S5 and 6.

**Table 4:** Antiviral activities of the aerial flowering extracts of *R. tuberosa* and *R. patula*.

	HAdV-40			H1N1			HSV-2		
	CC_50_^a^	IC_50_^a^	SI	CC_50_^a^	IC_50_^a^	SI	CC_50_^a^	IC_50_^a^	SI
Standard (Gallic acid)	8.342 ± 0.64	0.789 ± 0.03	10.56	–	–	–	–	–	–
Standard (Oseltamivir)	–	–	–	123.23 ± 3.26	0.865 ± 0.03	142.46	–	–	–
Standard (Acyclovir)	–	–	–	–	–	–	351.85 ± 6.79	29.04 ± 5.67	12.1
*R. tuberosa*	190.188 * ± 9.02	20.65 * ± 1.08	9.21	80.00 * ± 4.09	13.13 * ± 1.43	6.08	187.076 * ± 7.04	22.59 * ± 1.08	8.28
*R. patula*	218.718 * ± 6.05	32.26 * ± 1.57	6.78	129.521 * ± 6.53	23.03 * ± 1.13	5.63	107.913 * ± 2.35	11.66 * ± 1.13	9.26

*A significant difference between the investigated extracts and standard drugs at P < 0.5

### Computational analysis

#### Docking and molecular interaction studies with human adenovirus type 40

The cysteine protease adenine is the essential protease of adenovirus and, as such, represents a promising target for the treatment of ocular and other adenoviral infections. According to the docking results, the protease strongly attracted ellagic acid, quercetin, rutin, naringenin, hesperetin and kaempferol, with binding energies of − 7.20, − 6.70, − 6.90, − 6.20, − 6.90, and − 6.70 kcal/mol, respectively, compared with those of standard gallic acid (− 5.00 kcal/mol). These compounds formed hydrogen bonds with essential residues such as Asn44, Glu5, Gly51, Gln6, Ser3, Val53, Ser119, and Gln115, as well as hydrophobic interactions with (Pi-alkyl) Arg48, Ala120, Cys122, and Leu201; (Pi-Pi-stacked) Trp55; (C-Hydrogen bond) Gly52, Ser4, Gly51, and Val53; and (Pi-Cation) Gly2. Overall, these findings suggest that ellagic acid is the most promising compound for further study as a potential protease of adenovirus inhibitors. (Fig. 1 and Supplementary Table S1).

#### Docking and interaction studies with proteases of Herpes simplex virus type

**Figure 2:**
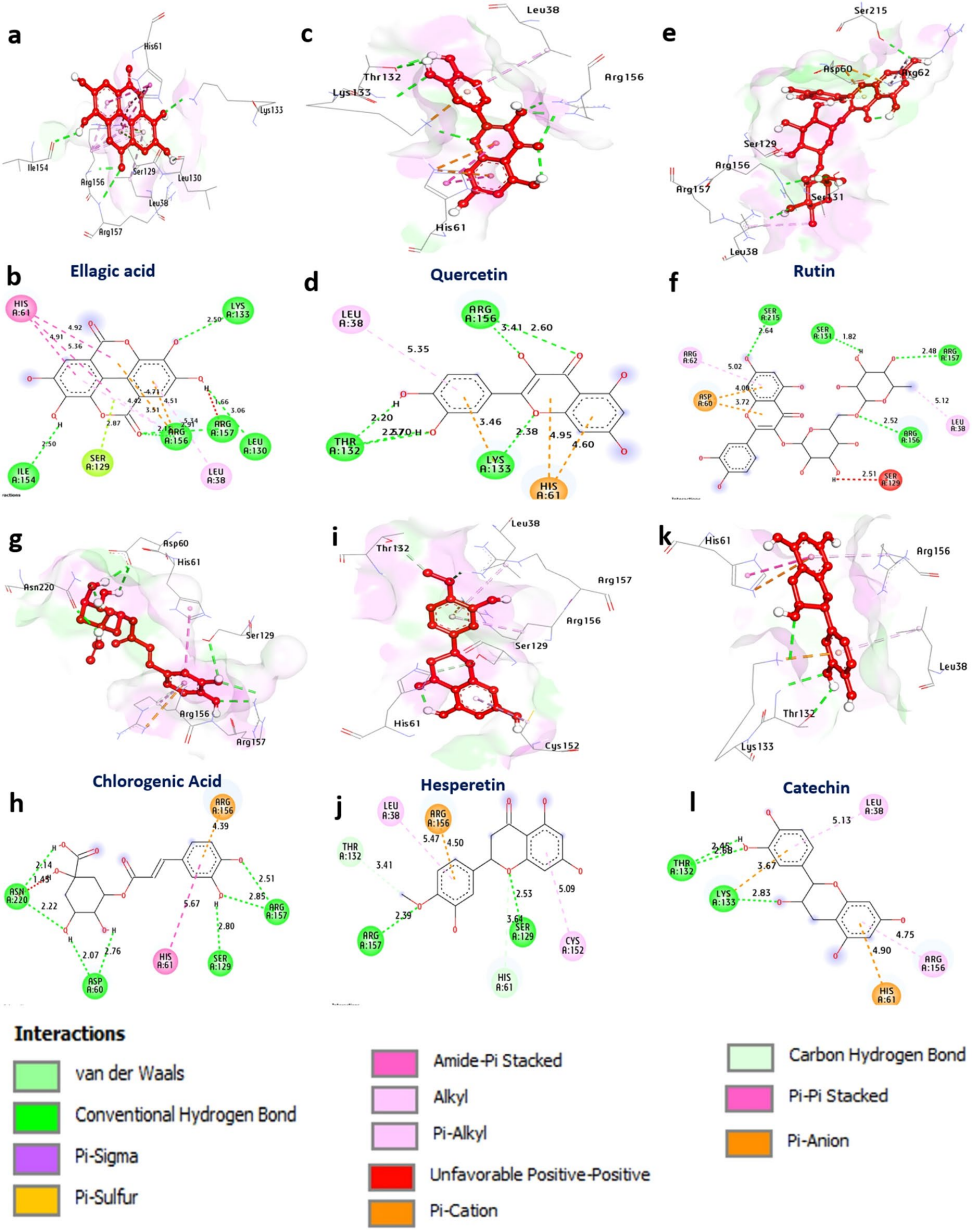
3D representations of compound conformations at the binding pocket of human Herpes simplex virus type 2 (PDB: ID 1AT3): (**a**,**b**) ellagic acid, (**c**,**d**) quercetin, (**e**,**f**) rutin, (**g**,**h**) chlorogenic acid, (**i**,**j**) hesperetin, and (**k**,**l**) catechin.

These proteases play important roles in HSV-2 replication and pathogenesis. According to the docking results, the protease enzyme appears to have a strong affinity for ellagic acid, quercetin, rutin, chlorogenic acid, hesperetin and catechin, with binding energies of − 6.30, − 6.30, − 7.90, − 7.00, − 6.60 and − 6.40 kcal/mol, respectively, compared with that of acyclovir as a standard (− 5.30 kcal/mol). These compounds formed hydrogen bonds with Leu130, Lys133, Arg157, Arg156, Ile154, Thr132, Ser131, Ser215, Ser129, Asp60 and Asn220. Additionally, hydrophobic interactions (Pi-alkyl) with Cys152, Leu38, Leu513, Arg156, and Arg62; (Pi-Cation) with Arg156, Lys133, His61 and Arg156; (Carbon H-Bond) with His61 and Thr132; (Pi-Pi T-shaped) with His61; (Pi-Pi stacked) with Arg156; (Pi-Lone Pair) with Ser129; and (Pi-Anion) with Asp60 were formed. The amino acids Thr132, Leu130, Ser129, and Arg156 in the catalytic site appeared to enhance the binding affinity of the compounds. Overall, these findings suggest that phenolic and flavonoid compounds, including chlorogenic acid, have strong potential as inhibitors of Herpes simplex virus type 2 proteases. (Fig. 2 and Supplementary Table S2).

#### Docking and interaction studies of influenza virus (H1N1) with neuraminidase

**Figure 3:**
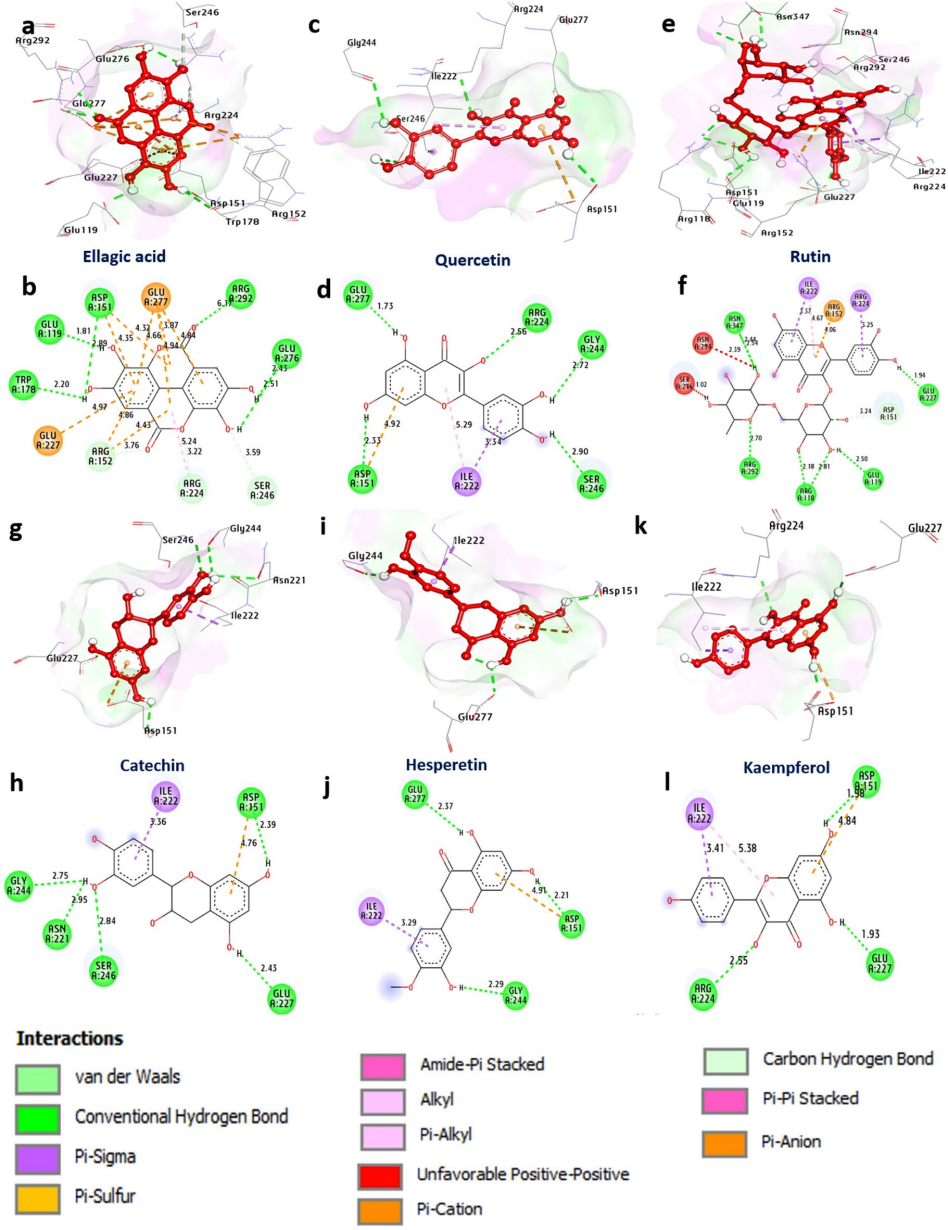
3D representations of compound conformations at the binding pocket of neuraminidase of influenza virus (H1N1) (PDB: ID 3B7E). (**a**,**b**) Ellagic acid, (**c**,**d**) quercetin, (**e**,**f**) rutin, (**g**,**h**) catechin, (**i**,**j**) hesperetin, (**k**,**l**) kaempferol.

Neuraminidase (NA) is an enzyme found on the surface of influenza viruses, specifically those belonging to the Orthomyxoviridae family. It plays a crucial role in the viral replication and release process. The docking results suggest that ellagic acid, quercetin, rutin, catechin, hesperetin and kaempferol have the greatest affinity for neuraminidase, with binding energies of − 8.20, − 8.60, − 8.90, − 8.20, − 8.20 and − 8.10 kcal/mol, respectively, compared with that of standard oseltamivir (− 6.30 kcal/mol). These compounds formed hydrogen bonds with Glu276, Arg292, Asp151, Glu119, Trp178, Arg224, Ser246, Arg118, Asn347, Glu227, Asn221, Glu277 and Gly244. Additionally, hydrophobic interactions (Pi-alkyl) with Arg224 and Ile222, (Carbon‒Hydrogen bond) with Arg224, Ser246, Arg152 and Asp151, (Pi-Sigma) with Ile222 and Arg224, (Pi-Anion) with Glu227, Asp151 and Arg152, and (Pi-Cation) with Glu277 formed. The amino acids Ile222, Gly244, Arg118, and Asp151 in the catalytic site appeared to enhance the binding affinity of the compounds. Overall, these findings suggest that rutin and quercetin may be promising candidates for further study as potential neuraminidases of influenza virus inhibitors (Fig. 3 and Supplementary Table S3).

#### In silico pharmacokinetic ADME prediction of synthesized compounds

**Figure 4:**
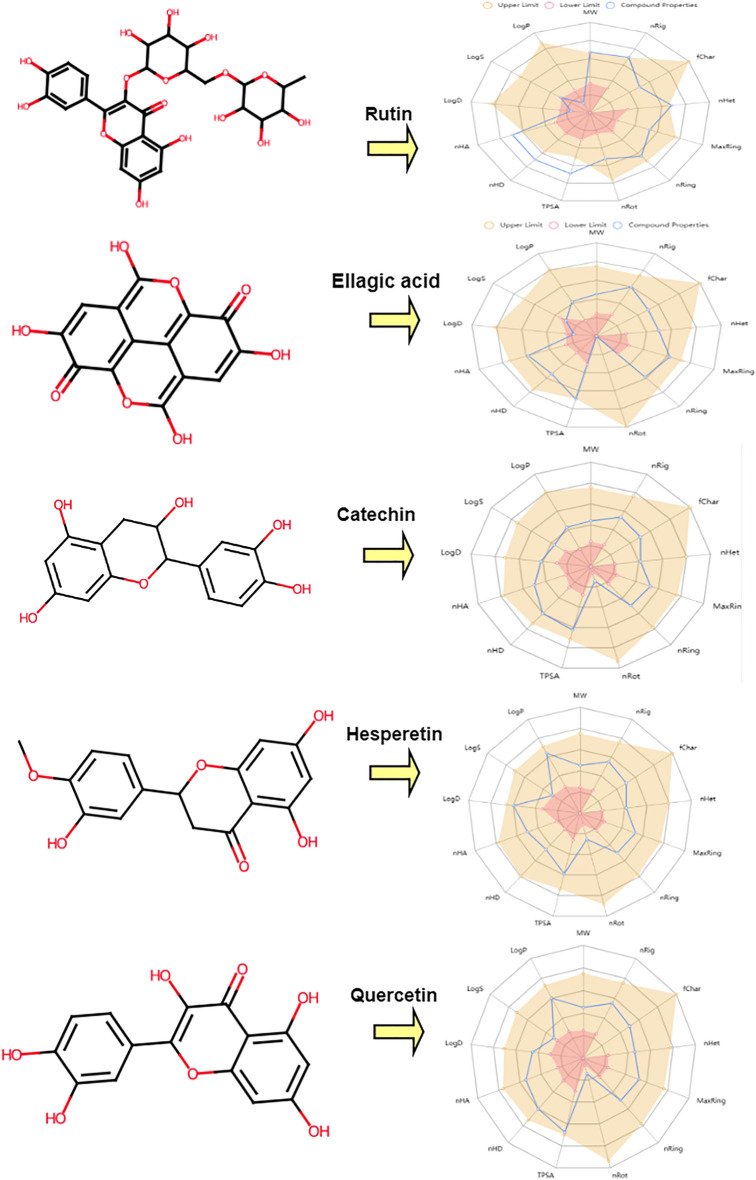
Oral bioavailability of the compounds determined with ADMETlab 2.0. Here, *MW* Molecular weight, *nRig* number of rigid bonds, *fChar* formal charge, *nHet* number of heteroatoms, *MaxRing* number of atoms in the largest ring, *nRing* number of rings, *nRot* number of rotatable bonds, *TPSA* topological polar surface area, *nHA* number of hydrogen bond acceptors, *nHA* number of hydrogen bond donors, *logS* Log of the aqueous solubility, *logP* Log of the octanol/water partition coefficient and *logD* logP at physiological pH 7.4.

Based on the docking results, the most promising compounds, rutin, ellagic acid, catechin, hesperetin, and quercetin, with the highest affinity for ADME and toxicity risks were identified. First, the physiochemical properties of the tested compounds are shown in Table 5 and Fig. 4. All physiochemical criteria, such as (MW) molecular weight; (nRig) number of rigid bonds; (fChar) formal charge; (nHet) number of heteroatoms; (MaxRing) number of atoms in the largest ring; (nRing) number of rings; (nRot) number of rotatable bonds; (TPSA) topological polar surface area; (nHA) number of hydrogen bond acceptors; and (nHA) number of hydrogen bond donors, were examined and evaluated. Therefore, all the compounds possessed enough rotatable bonds (RBs 4), which is crucial for high structural flexibility. This is important because compounds with fewer than ten RBs are more likely to be bioavailable. As the number of RBs increases, they become more critical in determining successful interactions with certain binding sites. The hydrogen bond acceptors (HBAs) and donors (HBBDs) were also calculated for the three compounds, and it was found that all compounds had less than 10 HBA and less than 5 HBD, indicating a favorable balance of HBA and HBD and a greater likelihood of oral bioavailability. Additionally, the TPSA was evaluated as a metric for assessing drug transfer characteristics. The TPSA values of the compounds were found to be relatively high, with most falling in the optimal range of 60–140 for good absorption in the gut and oral bioavailability. Second, the lipophilicity and water solubility of the compounds rutin, ellagic acid, catechin, hesperetin, and quercetin were assessed. The obtained findings indicated that all the active compounds are highly soluble in water and have moderate solubility. The Log S values of rutin, ellagic acid, catechin, hesperetin, and quercetin ranged from − 4.666 to − 2.99, indicating high water solubility. The presence of soluble molecules simplifies the synthesis, handling, and formulation of bioactive substances. Additionally, the lipophilicity parameter LOGP of all the compounds appeared to fall within the allowed range of LOGP values between − 0.763 and 2.473. Third, tests were also conducted on the pharmacokinetics of the compounds. The obtained results suggest that the examined compounds have high theoretical bioavailability and may be considered potential drug-like agents. However, all the compounds exhibited moderate intestinal absorption. Additionally, they have the potential to interact with other drugs because they can suppress the enzymes CYP1A2, CYP2C19, CYP2C9, CYP2D6, and CYP3A4. Fourth, the study appears to have evaluated the drug-likeness of compounds using various methods, including the Lipinski and Pfizer rules. Encouragingly, all the compounds met the drug-likeness requirements of Lipinski, indicating that they have desirable physicochemical properties for drug development and were rejected by Pfizer and the Golden Triangle rules. Regarding the distribution of compounds, many parameters, including plasma protein binding (PPB), were assessed. All compounds were present in more than 75% of the samples, with high protein-bound plasms having a low therapeutic index and a low fraction of unbound plasms (low: < 5%; middle: 5 ~ 20%; high: > 20%). Additionally, the blood–brain barrier (BBB) penetration of all compounds was calculated as the BBB, which cannot cross the blood–brain barrier. The volume distributions of all the compounds were obtained in the allowed range (0.04–20 L/kg). Finally, based on the computational assessment, it appears that the compounds are relatively safe and nontoxic (Table 6).

**Table 5:** Prediction of the pharmacokinetics and physicochemical properties of the compounds.

Id	ID	Rutin	Ellagic acid	Catechin	Hesperetin	Quercetin	Id	ID	Rutin	Ellagic acid	Catechin	Hesperetin	Quercetin
Physicochemical properties	MW	610.15	302.01	290.08	302.08	302.04	Metabolism	CYP1A2-inh	0.013	0.788	0.219	0.912	0.943
	Vol	552.318	265.705	279.249	293.909	282.767		CYP1A2-sub	0.026	0.088	0.295	0.857	0.115
	Dense	1.105	1.137	1.039	1.028	1.068		CYP2C19-inh	0.011	0.013	0.037	0.722	0.053
	nHA	16	8	6	6	7		CYP2C19-sub	0.05	0.043	0.056	0.063	0.041
	nHD	10	4	5	3	5		CYP2C9-inh	0.002	0.233	0.218	0.792	0.598
	TPSA	269.43	141.34	110.38	96.22	131.36		CYP2C9-sub	0.246	0.098	0.838	0.923	0.643
	nRot	6	0	1	2	1		CYP2D6-inh	0.007	0.006	0.173	0.605	0.411
	nRing	5	4	3	3	3		CYP2D6-sub	0.155	0.123	0.41	0.678	0.205
	MaxRing	10	14	10	10	10		CYP3A4-inh	0.013	0.053	0.315	0.815	0.348
	nHet	16	8	6	6	7		CYP3A4-sub	0.003	0.011	0.215	0.201	0.046
	fChar	0	0	0	0	0	Excretion	CL (Clearance)	1.349	2.346	17.911	15.68	8.284
	nRig	30	21	17	18	18		T12	0.524	0.863	0.853	0.773	0.929
	Flex	0.2	0	0.059	0.111	0.056	Toxicity	hERG Blockers	0.017	0	0.022	0.049	0.099
	nStereo	10	0	2	1	0		H-HT	0.092	0.144	0.071	0.112	0.1
Solubility	LogS	− 3.928	− 4.666	− 2.99	− 3.975	− 3.671		DILI	0.982	0.989	0.109	0.895	0.98
	LogD	0.695	0.794	1.618	2.6	1.767		AMES Toxicity	0.805	0.38	0.522	0.174	0.657
	LogP	− 0.763	1.117	1.142	2.473	2.155		Rat Oral Acute Toxicity	0.05	0.45	0.467	0.711	0.065
	ESOL Log S	− 2.45	− 1.99	− 2.18	− 2.75			FDAMDD	0.014	0.198	0.082	0.58	0.31
	Ali Log S	− 6.111	− 6.210	− 7.400	− 7.321			Skin Sensitization	0.036	0.716	0.945	0.925	0.919
	Silicon-IT class	Soluble	Soluble	Soluble	Soluble	Soluble		Carcinogenicity	0.064	0.314	0.09	0.419	0.05
Drug-likeness	Lipinski Rule	Rejected	Accepted	Accepted	Accepted	Accepted		Eye Corrosion	0.003	0.009	0.003	0.004	0.007
	Pfizer Rule	Accepted	Accepted	Accepted	Accepted	Accepted		Eye Irritation	0.01	0.725	0.903	0.925	0.936
	Golden Triangle	Rejected	Accepted	Accepted	Accepted	Accepted		Respiratory Toxicity	0.015	0.067	0.107	0.752	0.072
Absorption	Pgp-inh	0.002	0	0.007	0.006	0.004	Toxicophore rules	Non-Genotoxic Carcinogenicity	0	2	0	0	0
	Pgp-sub	0.978	0.006	0.002	0.003	0.005		LD50_oral	0	0	0	0	0
	HIA	0.925	0.198	0.096	0.014	0.014		Genotoxic	0	4	0	0	0
	F (20%)	0.234	0.073	0.99	0.067	0.93		Sure ChEMBL	0	0	0	0	0
	F (30%)	0.999	0.981	0.999	0.98	0.997		Non-Biodegradable	2	1	1	1	1
	Caco-2	− 6.336	− 5.312	− 5.971	− 4.878	− 5.204		Skin Sensitization	8	1	9	10	8
	MDCK	2.97E–−05	1.11E−05	4.27E–−06	7.73E−06	7.69E–−06		Aquatic Toxicity Rule	2	0	2	0	0
Distribution	BBB	0.111	0.011	0.025	0.035	0.008	Medicinal chemistry	Toxicophores	2	2	2	1	2
	PPB	83.81%	78.23%	92.06%	95.31%	95.50%		QED	0.14	0.356	0.51	0.789	0.434
	VDss	0.754	0.83	0.661	0.673	0.579		Synth	4.783	3.683	3.344	2.912	2.545
	Fu	20.87%	23.96%	8.87%	6.13%	7.42%		Fsp3	0.444	0	0.2	0.188	0

**Table 6:** Prediction of toxicity risks and oral toxicity prediction results of compounds.

No	Ligand	Toxicity risks				Physicochemical properties					
		Mutagenic	Tumorigenic	Irritant	Reproductive	CLogP	Solubility	Molecular weight	TPSA	Drug likeness	Drug score
1	Catechin	(−)	(−)	(−)	(−)	1.51	− 1.76	290.0	110.3	1.92	0.87
2	Rutin	(−)	(−)	(−)	(−)	− 1.26	− 2.40	610.0	265.5	3.31	0.57
3	Ellagic acid	(−)	(−)	(−)	(−)	1.28	− 3.29	302.0	133.5	− 1.60	0.51
4	Hesperetin	(−)	(−)	(−)	(−)	1.13	− 1.70	302.0	96.22	0.58	0.79
5	Quercetin	(+)	(−)	(−)	(−)	1.18	− 1.66	302.0	131.3	-4.35	0.11

#### Molecular dynamics simulation (MDS)

**Figure 5:**
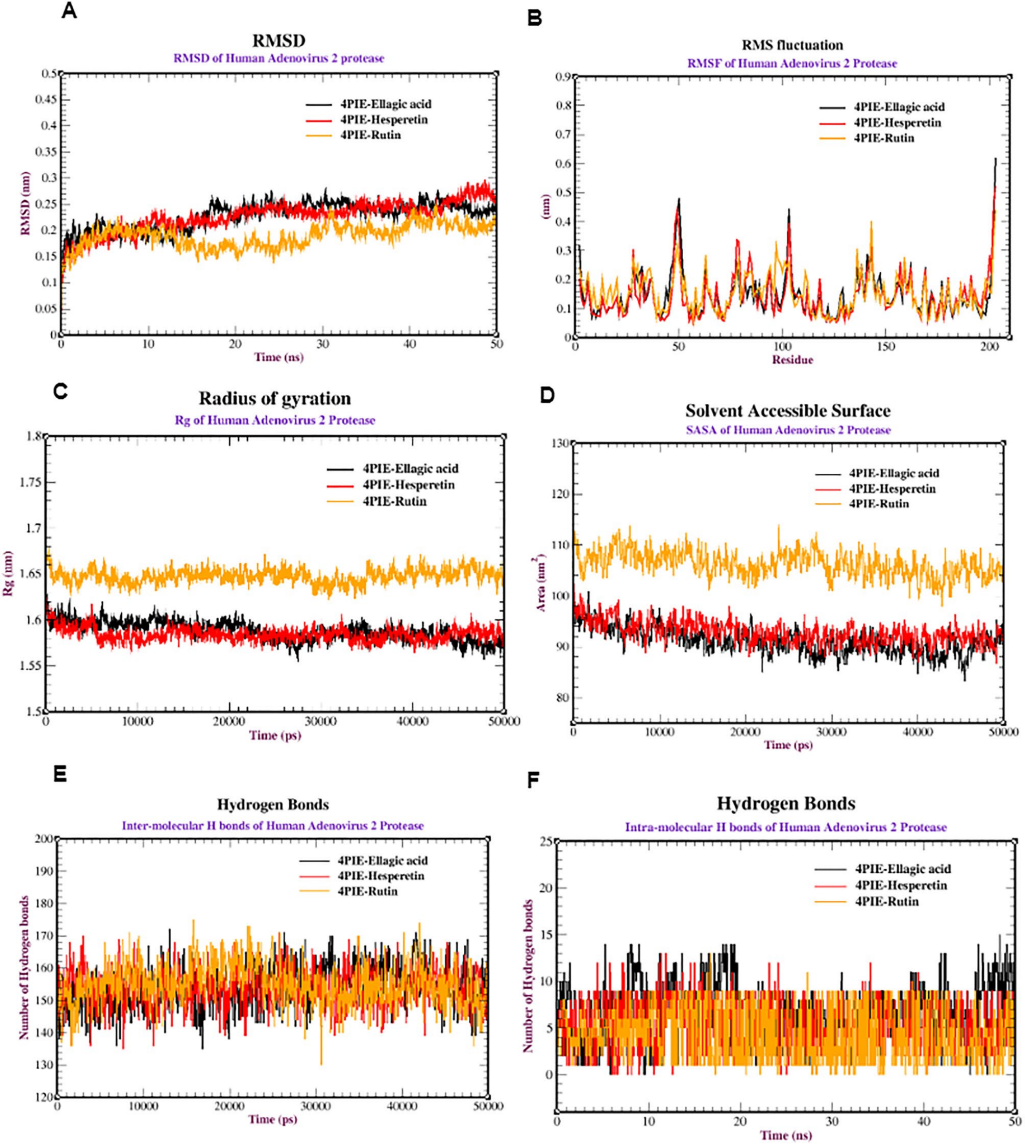
Molecular dynamics of cysteine proteases from human adenovirus type 40 (PDB:4PIE) complexed with ellagic acid, hesperetin, and rutin: (**A**) RMSD, (**B**) RMSF, (**C**) SASA, (**D**) radius of gyration (Rg), (**E**) intermolecular hydrogen bonds and (**F**) intramolecular hydrogen bonds.

Based on the docking of the three viral receptors with promising compounds, dynamic simulations were performed to investigate the behavior and stability of the protein complexes at the atomic level. First, several analyses of the MDS of a protease from human adenovirus (PDB: 4PIE) complexed with ellagic acid, hesperetin, and rutin were performed to assess the stability and dynamics of the 4PIE complexes. The RMSD was used to evaluate the stability of the protein structures. Figure 5A shows that 4PIE-Rutin was highly stable between 0.10 and 0.20 nm, while 4PIE-Hespertin and 4PIE-Ellagic acid exhibited stability between 0.10–0.25 nm and 0.15–0.25 nm, respectively. 4PIE-Rutin was stabilized after 30 ns, 4PIE-Hespertin after 20 ns, and 4PIE-Ellagic acid after 15 ns. Additionally, RMSF analysis was used to assess the flexibility of amino acid residues during the simulation (Fig. 5B). Most residues showed minimal fluctuations (0.1–0.3 nm), indicating relative stability. In addition, Rg analysis was performed to evaluate the overall shape of the protein complexes. Figure 5C shows Rg values ranging from 1.56 to 1.58 nm for 4PIE-Hespertin, 1.60–1.61 nm for 4PIE-ellagic acid, and 1.65–1.67 nm for 4PIE-rutin. The Rg values provide insights into the compactness or expansion of the protein structures during the simulation. Next, SASA analysis was conducted to understand the protein folding dynamics and stability. Figure 5D shows SASA values ranging from 85 to 95 nm for 4PIE-Hespertin, 88–95 nm for 4PIE-ellagic acid, and 105–115 nm for 4PIE-rutin. Furthermore, the intramolecular and intermolecular hydrogen bonds were analyzed to assess the stability of the complexes (Fig. 5E,F). The protease complexes with ellagic acid, hesperetin, and rutin initially formed a range of 140–170 intramolecular hydrogen bonds, which fluctuated during the simulation. In terms of intermolecular hydrogen bonds, ellagic acid formed the most interactions (2–14 bonds), followed by hesperetin (2–12 bonds) and rutin (2–8 bonds). These hydrogen bonds contribute significantly to the stability of the complex structures.

**Figure 6:**
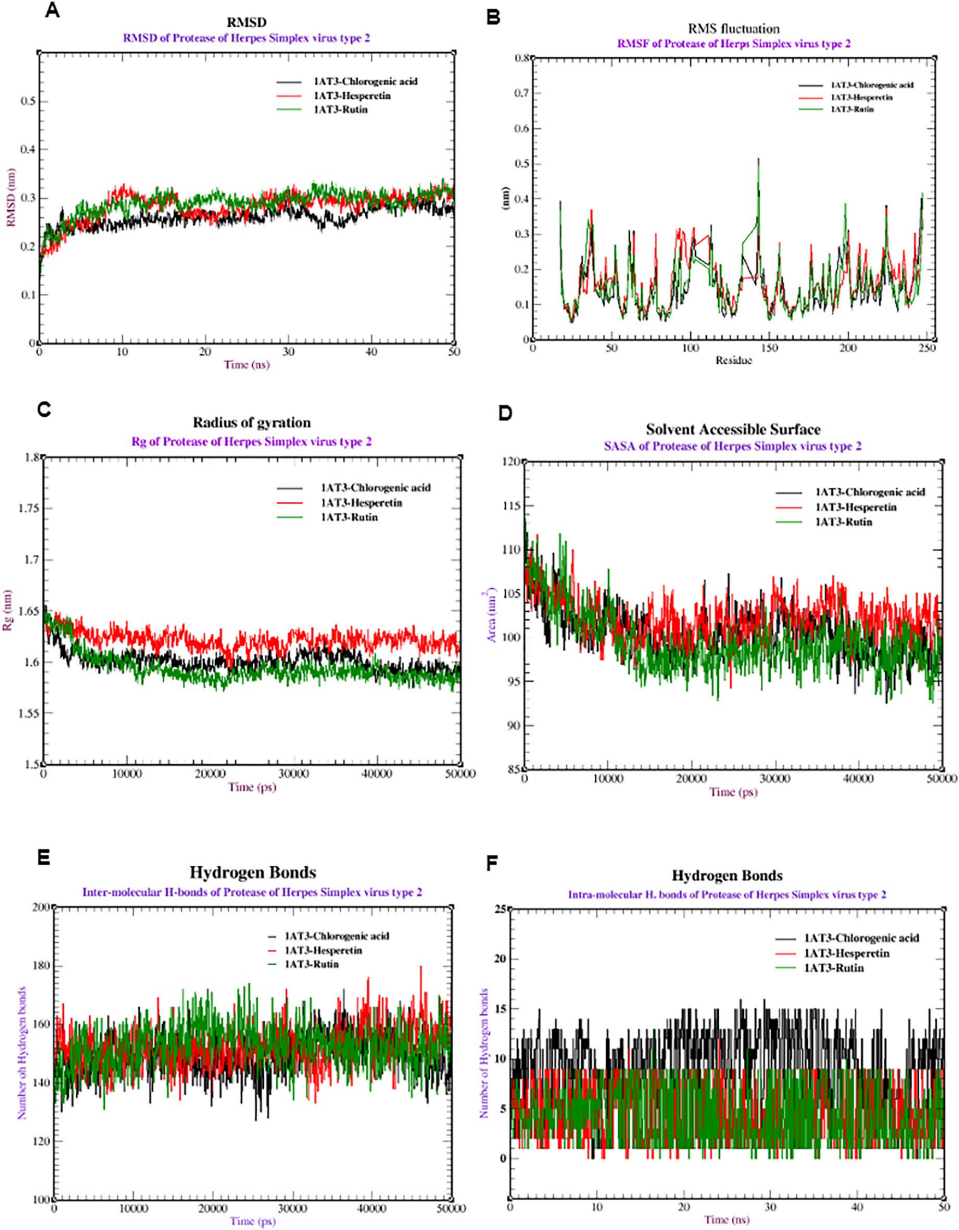
Molecular dynamics of proteases from Herpes simplex virus type 2 (PDB:1AT3) combined with chlorogenic acid, hesperetin, and rutin: (**A**) RMSD, (**B**) RMSF, (**C**) SASA, (**D**) radius of gyration (Rg), (**E**) intermolecular hydrogen bonds and (**F**) intramolecular hydrogen bonds.

Second, several MDS investigations were performed on Herpes simplex virus protease (1AT3) complexed with chlorogenic acid, hesperetin, and rutin to analyze the stability and dynamics of the complex. Figure 6A reveals that the RMSD values of 1AT3-chlorogenic were stable between 0.20 and 0.25 nm and stabilized after 10 ns, but those of 1AT3-Hespertin and 1AT3-rutin were stable between 0.20–0.30 nm and 0.18–0.28 nm and stabilized after 10 ns and 20 ns, respectively. Additionally, RMSF analysis was used to assess the flexibility of amino acid residues during the simulation (Fig. 6B). Most residues showed minimal fluctuations (0.1–0.3 nm), indicating relative stability. Figure 6C shows the Rg values for each complex, ranging from 1.60 to 1.63 nm for 4PIE-chlorogenic, 1.62 to 1.65 nm for 4PIE-Hesperetin, and 1.55 to 1.60 nm for 4PIE-rutin. Fourth, Fig. 6D shows the SASA values for each complex, ranging from 95 to 110 nm for 4PIE-chlorogenic and 4PIE-Hesperetin and from 93 to 105 nm for 4PIE-rutin. Figure 6E,F show that the number of intramolecular hydrogen bonds in the complexes with chlorogenic acid, hesperetin, and rutin initially ranged from 130 to 160 intramolecular hydrogen bonds, which fluctuated during the simulation. In terms of intermolecular hydrogen bonds, chlorogenic acid formed the most bonds (1–12 bonds), followed by hesperetin (2–10 bonds) and rutin (2–8 bonds).

**Figure 7:**
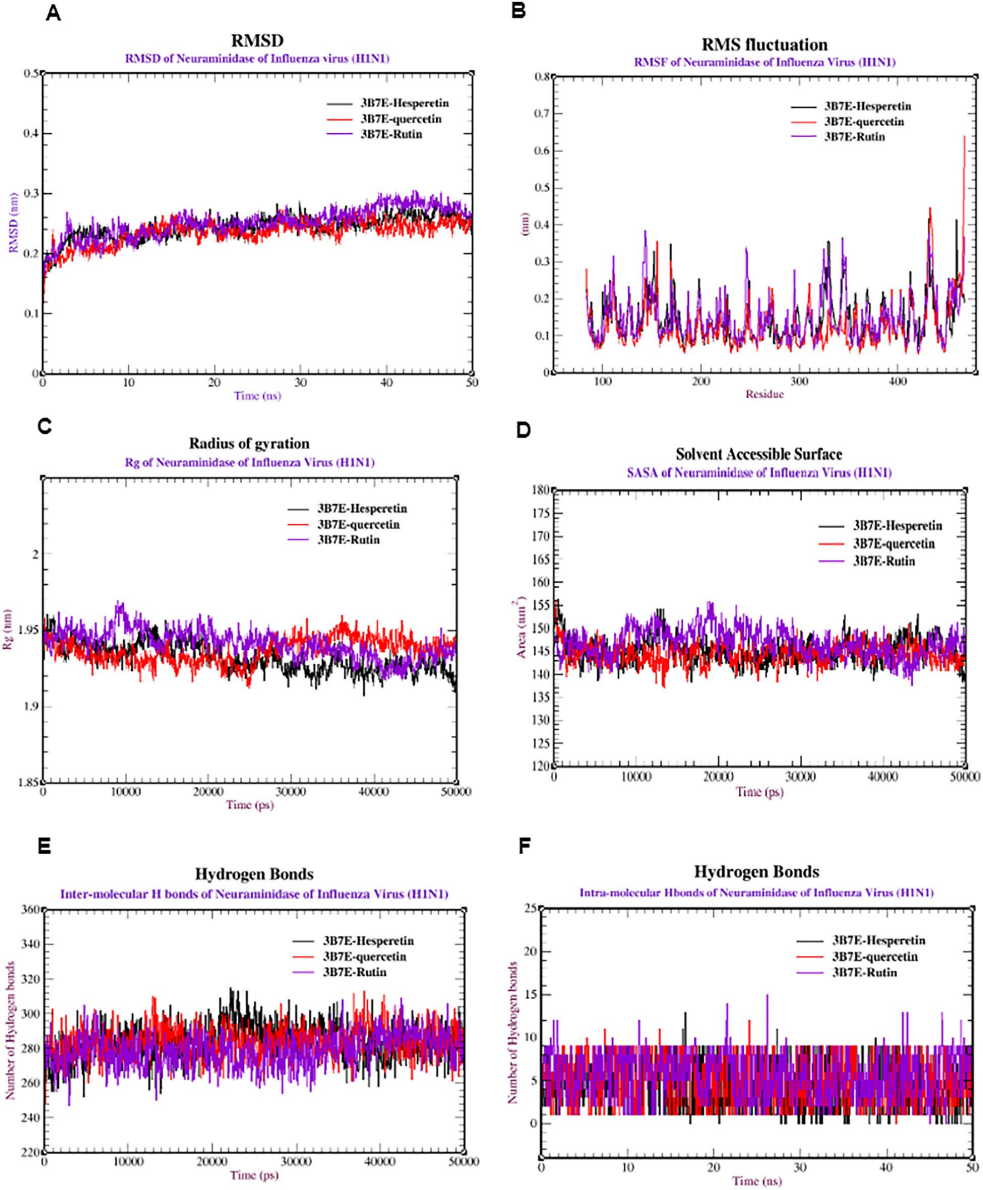
Molecular dynamics of the neuraminidase of influenza virus (H1N1) (PDB: 3B7E) complexed with quercetin, hesperetin, and rutin: (**A**) RMSD, (**B**) RMSF, (**C**) SASA, (**D**) radius of gyration (Rg), (**E**) intermolecular hydrogen bonds and (**F**) intramolecular hydrogen bonds.

Finally, several factors were investigated using dynamic simulations to determine the stability and dynamics of the H1N1 influenza virus neuraminidase protein (PDB: 3B7E) when attached to quercetin, hesperetin, and rutin. Figure 7A shows that 3B7E-quercetin was highly stable, with an RMSD ranging from 0.15 to 0.22 nm. 3B7E-Hesperetin and 3B7E-Rutin ranged from 0.20–0.25 nm and 0.20–0.30 nm, respectively. These complexes became stable after 15 ns for 3B7E-quercetin, 20 ns for 3B7E-hesperetin, and 15 ns for 3B7E-rutin. The majority of RMSF analyses showed minor variability, ranging from 0.1 to 0.35 nm, indicating that the protein complexes are relatively stable (Fig. 7B). Furthermore, the Rg values varied from 1.93 to 1.96 nm for 3B7E-quercetin, from 1.90 to 1.95 nm for 3B7E-hesperetin, and from 1.93 to 1.98 nm for 3B7E-rutin (Fig. 7C). Furthermore, Fig. 7D shows the SASA values ranging from 140 to 150 nm for 3B7E-quercetin, 140 to 155 nm for 3B7E-Hesperetin, and 135 to 150 nm for 3B7E-rutin. Finally, Fig. 7E,F depict the number of intramolecular and intermolecular hydrogen bonds formed by the complexes with quercetin, hesperetin, and rutin. Initially, the complexes formed a range of 250–300 intramolecular hydrogen bonds. Rutin formed the most intermolecular hydrogen bonds (12 bonds), followed by quercetin (2–8 bonds) and hesperetin (1–8 bonds).

## Discussion

The potential impacts of phenolic compounds on a range of ailments, such as cancer, heart problems, stroke, and inflammation, and their antibacterial and antiviral properties have aroused a great deal of interest in medicinal plants^78^. Because it offers higher sensitivity and selectivity than other LC techniques, LC–MS/MS has been applied extensively in quantitative applications for numerous medicinal plants^79–83^. The richness of polyphenols, especially flavonoids and phenolic acids, was revealed by LC–MS/MS analyses of both *Ruellia* extracts. These analyses led to the identification of 77 phenolic chemicals, which were divided into the following categories: 53 flavonoids, 13 phenolic acids and their derivatives, 3 stilbenes, 4 coumarins, and 5 catechol compounds. Flavonols, Flavonoid-3-*O*-glucuronides, Flavonoid glucuronides, Flavonoid-*O*-p-coumaroyl glycosides, isoflavonoid glycosides, isoflavonoids, methylated flavonoids, and anthocyanidins were among the various kinds of flavonoids that were found. This result is in line with earlier studies that found flavonoids and phenolic acids to be two of the most important secondary metabolites in *Ruellia* species^25^. Both plant extracts contained luteolin, apigenin, quercetin, and *p*-coumaric acid, as previously described^25^. The semipolar character of both *Ruellia* extracts makes them suitable for the presence of polyhydroxy flavonoid aglycones and flavonoid glycosides with sugar units. Table 1 displays the number of chemicals observed in each ionization mode: five in the positive mode and 47 in the negative mode. This highlights how crucial it’s to gather MS data across a broad spectrum of metabolites, ranging from basic to neutral and acidic, by employing both ionization techniques. More [M − H]^−^ ions are produced by the flavonoid glycosides than [M + H]^+^ ions. Their principal distinctive fragment ions resulting from the retroDiels Alder fragmentation pathway could be identified in their MS/MS spectra, along with losses of glycosyl moieties in both negative and positive ion modes. In terms of flavonoid glycosides, pentose (arabinose or xylose), rhamnose, hexose (glucose or galactose), and hexuronic or glucuronic acid were the common losses of 132, 146, 162, and 176 a.m.u., respectively. Additionally, flavonoids tended to be lost at 28 a.m.u. (CO), 18 a.m.u. (H_2_O), and 15 a.m.u. (CH_3_), indicating the presence of hydroxyl and methyl groups from phenolic compounds and facilitating the identification of flavonoid subgroups^48,61^. Seven distinct parent ion peaks can be found in both the positive and negative ion modes: 577.16 [M − H]^−^, 301.1 [M − H]^−^, 463.01 [M − H]^−^, 597.51 [M + H]^+^, 477.09 [M − H]^−^, and 269.05 [M − H]^−^. These peaks correspond to the respective compounds Rhoifolin, Hesperetin, Isoquercitrin, Eriodictyol-7-*O*-neohesperidoside, Quercitrin, and Apigenin (Table 2). Moreover, kaempferitrin, kaempferol-3-glucuronide, kaempferol-3-*O*-(6-p-coumaroyl)-glucoside, kaempferol-3-*O*-glucoside, kaempferol-3-*O*-alpha-l-arabinoside, kaempferol-3-*O*-alpha-l-rhamnoside, kaempferide, and kaempferol are the eight characteristic parent ion peaks for kaempferol and its derivatives and glycosides (Table 2). In addition, 8 parent ion peaks for quercetin-3-glucuronide, quercetin-3-*O*-arabinoglucoside, quercetin-3,4′-*O*-di-beta-glucopyranoside, quercetin, quercetin-3-d-xyloside, quercetin-7-*O*-rhamnoside, quercetin-3-arabinoside, and quercitrin were observed at 477.35 [M − H]^−^, 595.49 [M − H]^−^, 625.50 [M − H]^−^, 301.03 [M − H]^−^, 433.08 [M − H]^−^, 447.09 [M − H]^−^, 433.08 [M − H]^−^, and 447.09 [M − H]^−^, respectively. Luteolin was tentatively assigned based on its parent ion at *m/z* = 285 and fragment ions at *m/z* = 151 (ring A) and *m/z* = 133 (ring B) after ring C cleavage. Apigenin was identified at *m/z* = 269 with a characteristic fragment ion at *m/z* = 117 (ring B) and *m/z* = 151 (ring A). Luteolin-3′7-di-*O*-glycoside was tentatively identified by its molecular ion at *m/z* = 609 and two characteristic fragment ions at *m/z* = 447 (loss of one hexose molecule) and *m/z* = 285 (aglycone). Apigenin-8-C-glycoside (vitexin) with a molecular ion at *m/z* = 431 and a fragment ion at *m/z* = 269 (loss of hexose molecule) and fragment ion of the aglycone, along with two characteristic fragment ions at *m/z* = 341 ([M-H-90]^−^) and *m/z* = 311 ([M-H-120]^−^). Rhoifolin was identified with a molecular ion at *m/z* = 577 and a fragment ion at *m/z* = 269 ([M-H-Rham-Glc]^−^). A methoxy flavone, Acacetin (4′-*O*-methylated flavone) was identified at *m/z* = 283, with fragment ions at *m/z* = 268, 239, and 211 after the loss of CH3, CO_2_, and CO, respectively. Baicalein-7-*O*-glucuronide (trihydroxyflavone) was identified with a molecular ion at *m/z* = 445 and a baicalein fragment ion at *m/z* = 269 ([M-H-176]^−^, loss of glucuronide moiety). Quercetin (flavon-3-ol) was identified by its molecular ion at *m/z* = 301. Quercetin-3-d-xyloside is characterized by its molecular ion at *m/z* = 433 and the same aglycone peak at *m/z* = 301. Kaempferol-3-glucuronide by its molecular ion at *m/z* = 461 and the characteristic loss of glucuronic acid moiety (176), leaving an aglycone peak at *m/z* = 285. Isorhamnetin-3-*O*-glucoside by its molecular ion at *m/z* = 477 and fragment ions at *m/z* = 315 ([M-H-(Glc-H2O)-CH3]^−^), *m/z* = 300 ([M-H-(Glc-H2O)-CH3-CO]^−^), and *m/z* = 270; and isorhamnetin-3-*O*-rutinoside identified by its molecular ion at *m/z* = 623, with fragment ions at *m/z* = 477 ([M-H-146]^−^), *m/z* = 461 ([M-H-162]^−^), and the aglycone at *m/z* = 315. Syringetin-3-*O*-galactoside (*O*-methylated-flavonol) was detected with a molecular ion at *m/z* = 509 and the aglycone at *m/z* = 347. Daidzein-8-C-glucoside was found with a molecular ion at *m/z* = 415, a fragment ion at *m/z* = 295 characteristic of C-glycoside ([M-H-120]^−^), and the aglycone at *m/z* = 253 ([M-H-160]^−^). Naringenin was identified by its molecular ion at *m/z* = 271 and fragment ions at *m/z* = 151 (ring A) and *m/z* = 119 (ring B). Hesperetin-7-*O*-neohesperidoside was detected with a molecular ion at *m/z* = 609 and a fragment ion of the aglycone hesperetin at *m/z* = 301, indicating the loss of neohesperidose ([M-308]^−^)^84^. Phenolics are a group of secondary metabolites with different types of promising biological activities^85^. Phenolic acids are commonly reported metabolites in most profiling studies of medicinal plants. Phenolic acids generally produce the precursor ion [M − H]^−^, corresponding to deprotonated molecules, and the fragment ion [M‑H‑44]^−^, corresponding to the loss of CO_2_ from the carboxylic acid group^86^. In this study, 13 phenolic acids were identified, including gallic acid, d-(−)-quinic acid, coumaric acid, 3,4-hydroxybenzoic acid, vanillin, methyl gallate, p-coumaric acid, syringic acid, ferulic acid, chlorogenic acid, gentisic acid, ellagic acid, and caffeic acid, at [M − H]^−^ values of 168.01, 191.05, 163.04, 137.02, 151.04, 183.03, 163.04, 197.04, 193.05, 353.09, 153.02, 301.2, and 179.15, respectively. *P*-coumaric acid appeared with a molecular ion at *m/z* = 163 and a fragment ion at *m/z* = 119 (quinic acid—CO_2_). Chlorogenic acid was recognized by its deprotonated molecule [M−H]^−^ at *m/z* = 353 and a fragment ion at *m/z* = 191, corresponding to deprotonated quinic acid. The quinic acid molecular ion (*m/z* = 191) was also detected. The caffeic acid molecular ion was identified at *m/z* = 179, with a fragment ion at *m/z* = 135 (caffeic acid—CO_2_)^84^.

Catechins were observed at [M − H]^−^ values of 109.03, 289.07, and 289.07 for pyrocatechol, (−)-epicatechin, and catechin, respectively. Stilbenes such as *E*-4,5′-dihydroxy-3-methoxy-3′-glucopyranosylstilbene, *E*-3,4,5′-trihydroxy-3′-glucopyranosylstilbene and resveratrol were observed at [M − H] ^−^ values of 419.39, 405.39, and 227.07, respectively. Four coumarins, 7-hydroxy-4-methyl coumarin, rosmarinic acid, esculetin, and esculin, were observed at 175.04 [M−H]^−^, 359.1 [M − H]^−^, 177.02 [M − H]^−^, and 339.07 [M − H]^−^, respectively. Rosmarinic acid was identified by its molecular ion at *m/z* = 359, with a fragment ion at *m/z* = 197, representing the loss of caffeic acid. Esculetin (6,7-dihydroxy coumarin) was found with a molecular ion at *m/z* = 177 and a fragment ion at *m/z* = 133. Esculin was identified with a molecular ion at *m/z* = 339 and a fragment ion at *m/z* = 177 (loss of sugar)^84^. Flavonoids such as luteolin and quercetin, their glycosides, coumarins such as coumarin, and phenolics such as vanillic acid, p-coumaric acid, and syringin were previously reported^25^.

HPLC is the preferred analytical tool for fingerprinting and quantification of marker compounds in herbal drugs because of its simplicity, sensitivity, accuracy, suitability for thorough screening, etc.^87^. In our study, HPLC analysis was also conducted to quantitatively estimate the content of some phenolic compounds that were mentioned in the current LC-MSMS and present in the flowering aerial extracts of *R. tuberosa* and *R. patula*. HPLC analyses did not quantitatively detect Pyro catechol or quercetin in *R. tuberose* or catechin, caffeic acid, vanillin, daidzein, or hesperetin in *R. patula* despite being tentatively identified by LC–MS/MS analyses. This could be due to the ability of the LC–MS/MS technique to detect phenolic compounds at lower concentrations than the HPLC technique^88^. To the best of the authors’ knowledge, this is the first report on the detection of seventeen and fourteen phenolic compounds in the aerial extracts of *R. tuberosa* and *R. patula*, respectively, from flowering plants.

In this study, the floral aerial parts of *R. tuberosa* and *R. patula* were assessed for their potential for use as anti-adenoviral agents for the treatment of adenovirus infections. Among the extracts tested, *R. tuberosa* showed the most potent anti-ADV activity (IC_50_ of 20.65 µg/ml). Extracts of *R. patula* showed little activity against ADV (IC_50_ equal to 32.26 µg/ml). Previous studies have also reported that the genus *Ruellia* has antibacterial and antifungal activities^21–24^. Phenolics and flavonoids such as gallic acid, ellagic acid, quercetin, and their conjugates, which were found in our work and other publications using various chromatographic techniques, may be responsible for the actions of both *Ruellia* species under investigation^25,89^. It has been demonstrated that certain naturally occurring quercetin molecules have antiviral or antibacterial properties. Research has also demonstrated that quercetin functions similarly against ADV-3 and ADV41. Quercetin has also been shown in earlier research to have antiviral effects on HIV, HSV, ADV3, ADV8, and ADV11. According to previous studies, the EC_50_ values of pure substances for all anti-infective bioassays should be less than 25 mM^89^.

Numerous viruses, such as dengue virus, hepatitis B virus, herpes simplex virus, respiratory syncytial virus, parainfluenza virus, and adenovirus, were susceptible to the antiviral effects of flavonoids. It has been previously shown that these plant components interact with intracellular phases of the viral replication cycle in certain viruses. Regarding rotavirus, the glycone form of flavonoids works better than the aglycone form of these flavonoids. The flavonoid fraction had an inhibitory effect on the HSV-1 replication cycle and was effective against both HSV-1 and HSV-2. Our results may therefore suggest that the flavonoid concentrations of the aerial extracts of *R. tuberosa* and *R. patula*, which have promising anti-HSV-2 activity, are the cause of these findings^90^.

Phenolic compounds were used as sources of inspiration for the development of novel antiviral medications due to their antiviral efficacy against a variety of viruses. Recent studies have focused on the possible antiviral qualities of phenolic chemicals present in both *Ruellia* species. Numerous phenolic compounds found in *Ruellia* species have been demonstrated through independent studies to display antiviral action by inhibiting H1N1. *R. tuberosa* and *R. patula* showed anti-H1N1 viral activity at IC_50_s of 13.13 and 23.03 µg/ml, respectively, for both *Ruellia* species. Because they can interact with and inhibit the proteins and enzymes of both the virus and the host, phenolic substances have antiviral properties that prevent viral reproduction and infectivity^77^. Many studies have examined the antiviral properties of more than 100 selected phenolic compounds, among the numerous research groups studying the viral inhibitory effect of these compounds. These studies concluded that phenolic compounds with a high number of (–OH) groups and specific positions had the greatest inhibitory effect. Numerous substances possessing inhibitory properties against viruses have been identified in plants commonly used as traditional medicinal herbs or cuisines^91^. Chen et al. examined the bioavailability of phenolic compounds and discovered that, due to the high bacterial conjugative enzyme activity in the gastrointestinal tract environment, free phenolic compounds, such as quercetin, were assumed to partially degrade through dehydration reactions to hydroxybenzoic acids through gastric absorption^91^. Many of the phenolic compounds can be supplied through the colon, avoiding breakdown in the small intestine, and larger amounts can be found in the blood plasma by keeping the phenolic compounds bonded in a dietary matrix. Nevertheless, this adsorption technique provides only untargeted, low quantities of phenolic chemicals^87^. Gan et al. investigated the pharmacokinetic plasma concentration–time profiles for the adsorption of phenolic compounds from an extract of *Echinacea purpurea* in a rat model. They discovered that the ingestion of chlorogenic and caffeic acid causes adsorption to occur in 15 and 360 min, respectively, with corresponding disappearance half-lives of 7.72 and 6.00 h^91^. For more than thirty years, computer-aided drug design, or CADD, has gained popularity as a technique for designing, developing, and screening therapeutically significant molecular candidates^92^. These techniques, which aid in the more efficient optimization and screening of chemicals, combine several methodologies, such as molecular docking, toxicity, ADME (absorption, distribution, metabolism, and excretion), and molecular dynamics (MD) simulation. Currently, molecular docking is another well-liked technique that aids in determining how small molecule candidates interact with a target protein^91^. As a result, an in silico docking study was performed on the phenolic and flavonoid structures found in HPLC extracts of the aerial floral portions of *R. tuberosa* and *R. patula* against HADV-40, HSV-2, and H1N1 ^94^. The protease of HAdV-40 exhibited considerable binding affinity (− 7.20, − 6.90, and − 6.90 kcal/mol) when ellagic acid, rutin, and hesperetin were docked. Similarly, rutin, quercetin acid, and hesperetin showed affinities (− 8.90, − 8.60, and − 8.20 kcal/mol, respectively) for the H1N1 virus neuraminidase, whereas rutin, chlorogenic acid, and hesperetin showed affinities (− 7.90, − 7.00, and − 6.60 kcal/mol, respectively) for the HSV-2 protease. Additionally, the physicochemical characteristics and ADMET values of these potential compounds were computed. Additionally, MD simulations verified the stability of the complexes involving the neuraminidase of H1N1, proteases of HSV-2, and proteases of HAdV-40 (with RMSD of 0.10–0.25 nm, RMSF of 0.1–0.3 nm, SASA of 85–115, and Rg of 1.56–1.67 nm), proteases of HSV-2 (with RMSD of 0.18–0.30 nm, RMSF of 0.1–0.3 nm, SASA of 93–110, and Rg of 1.55–1.65 nm) and proteases of HAdV-40 (with RMSD of 0.10–0.25 nm, RMSF of 0.10–0.35 nm, SASA of 135–155, and Rg of 1.90–1.98 nm).

## Conclusion

This study focused on the phytochemical profiling and antiviral activities of extracts from *Ruellia* species. Both in vitro and in silico approaches were used to explain the potential antiviral effects of these extracts. In the in vitro analysis, we examined the phytochemical composition of the *Ruellia* species extracts. They identified and quantified various bioactive compounds present in both extracts, which provided insights into their potential antiviral effects. Additionally, through computational docking and molecular dynamics simulations, valuable information has been obtained regarding the molecular interactions between bioactive compounds and specific viral targets. The combined findings from the in vitro and in silico experiments provided a comprehensive evaluation of the antiviral activities of both *Ruellia* species extracts.

### Supplementary Information

Supplementary Information.

## Data Availability

All the data generated or analyzed during this study are included in this published article.
