# Supplementary material for: Phytochemical profiling, antiviral activities, molecular docking, and dynamic simulations of selected Ruellia species extracts
Source: Sci Rep. 2024 Jul 4;14:15381. doi: 10.1038/s41598-024-65387-5 (PMC11224336; doi:10.1038/s41598-024-65387-5)
Supplement: Supplementary file 1 — Supplementary Information. [file 41598_2024_65387_MOESM1_ESM.docx]

**Phytochemical profiling, antiviral activities, molecular docking, and dynamic simulations of selected *Reullia* species extracts**

**Mina Michael Melk^1^*, and Ahmed F. El-Sayed^2, 3^**

^1^ Pharmacognosy Department, Faculty of Pharmacy, Ahram Canadian University, Giza, Egypt; [mina.michael@acu.edu.eg](mailto:mina.michael@acu.edu.eg) (M.M.M.)

^2^Microbial Genetics Department, Biotechnology Research Institute, National Research Centre, Giza, Egypt; af.rizk@nrc.sci.e.g. (A.F.S.)

^3^Egypt Center for Research and Regenerative Medicine (ECRRM), Cairo, Egypt; ahmedfikry.nrc@gmail.com (A.F.S.)

*Correspondence: [mina.michael@acu.edu.eg](mailto:mina.michael@acu.edu.eg) (M.M.M.)

**Figure S1: LC-MS/MS chromatograms: A) *R. tuberosa* extract and B) *R. patula* extract.**

A

B

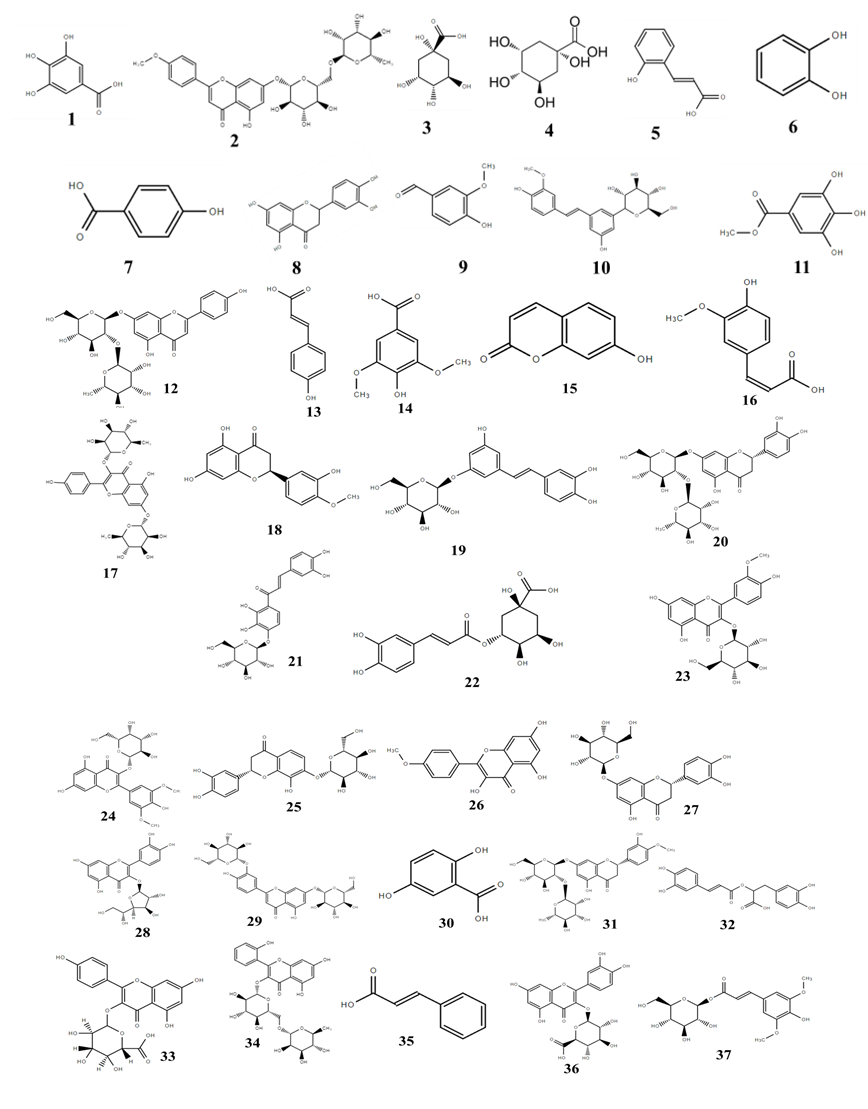


**Figure S2: Identified compounds from LC-MS/MS.**


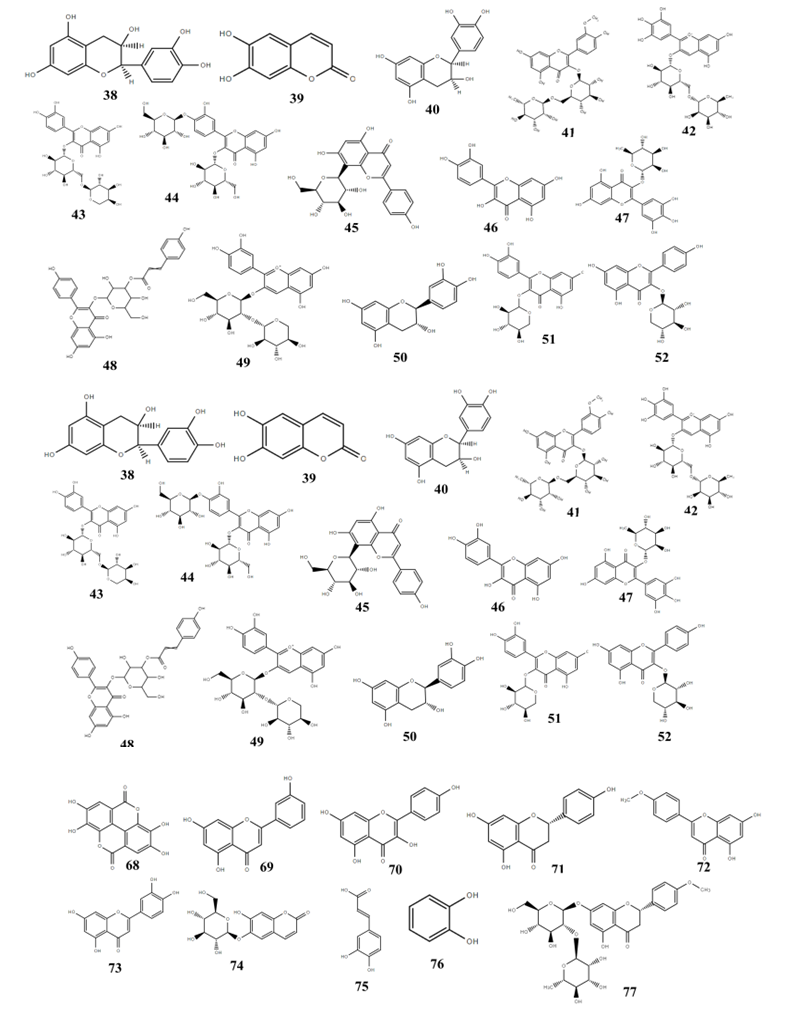


**Figure S3: Identified compounds from LC-MS/MS.**

**Figure S4: HPLC chromatograms: A) standard compounds, B) *R. tuberosa* extract, C) *R. patula* extract.**

C

B

A


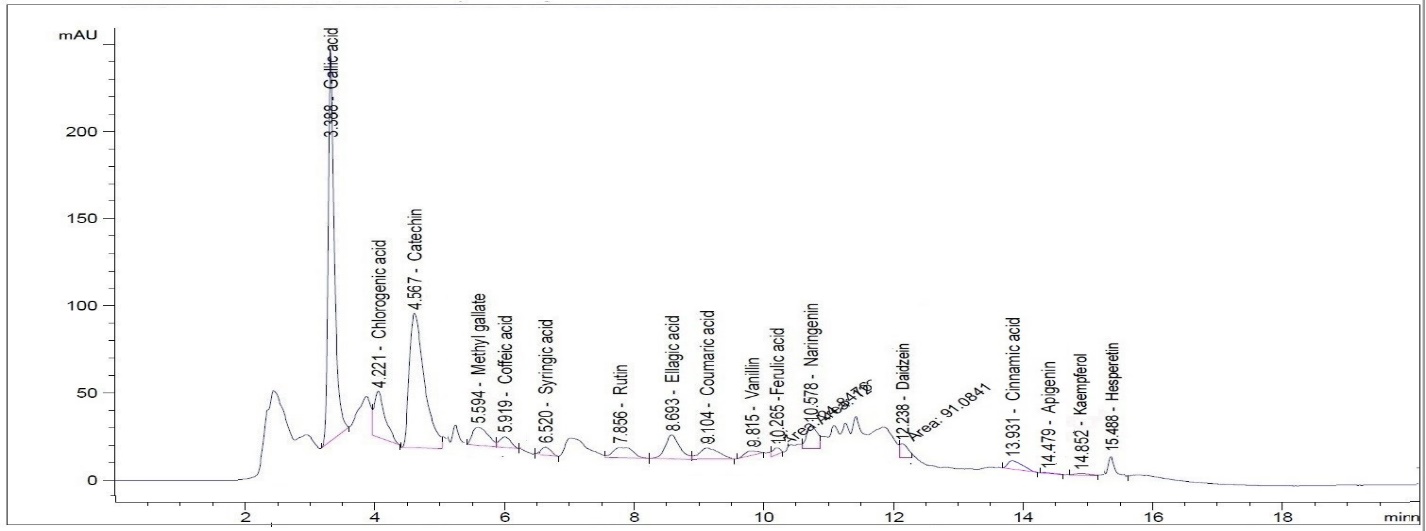

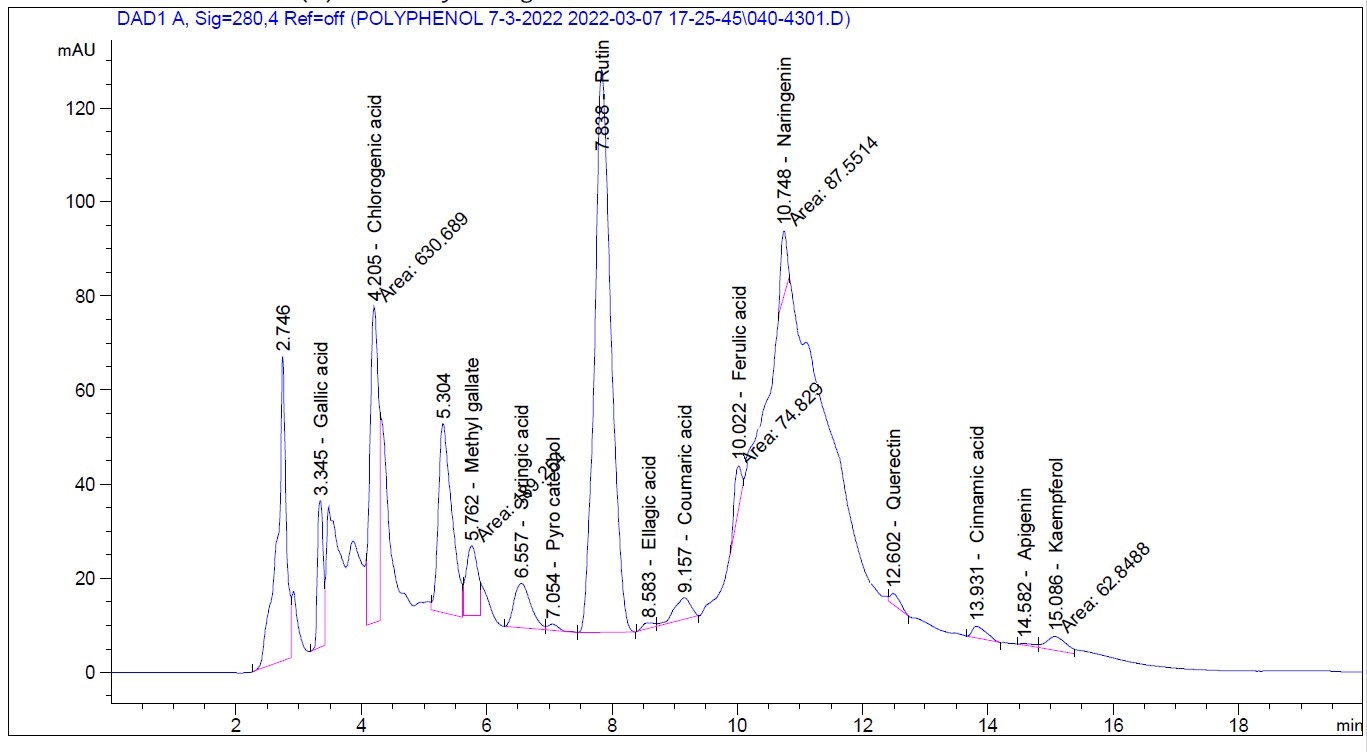

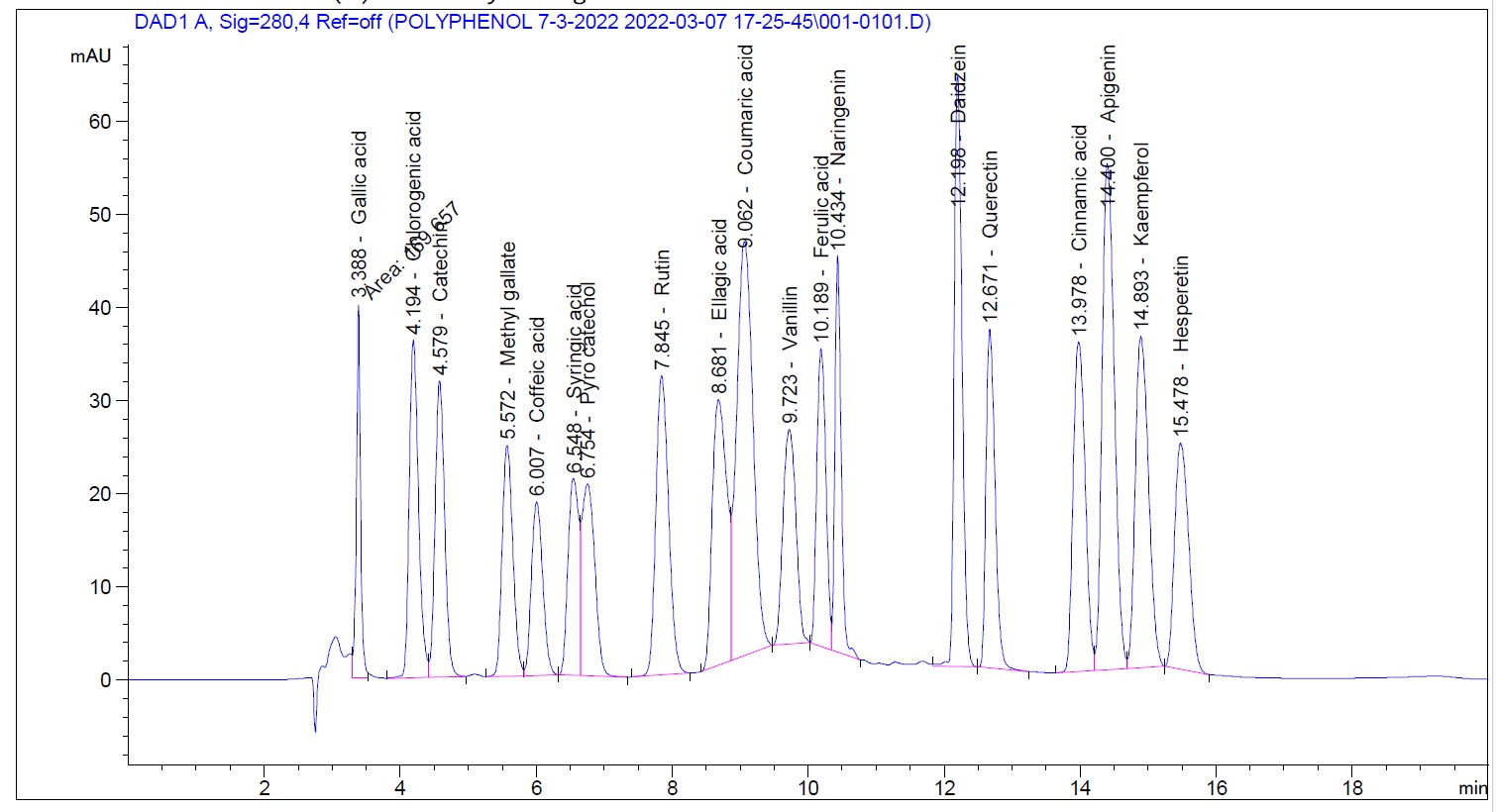


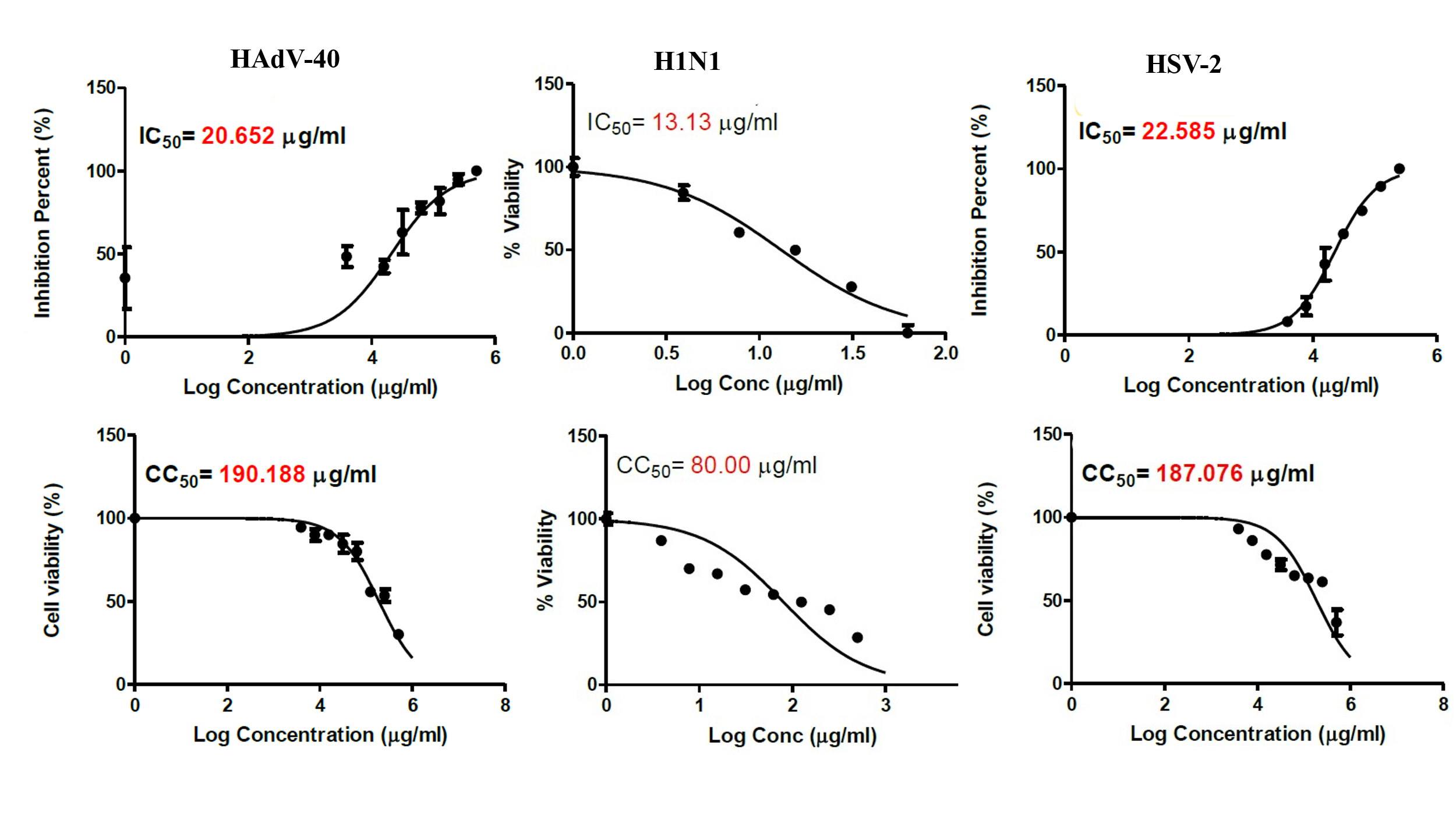


**Fig S5. Antiviral activities of the aerial flowering plant extracts of *R. tuberosa*.**


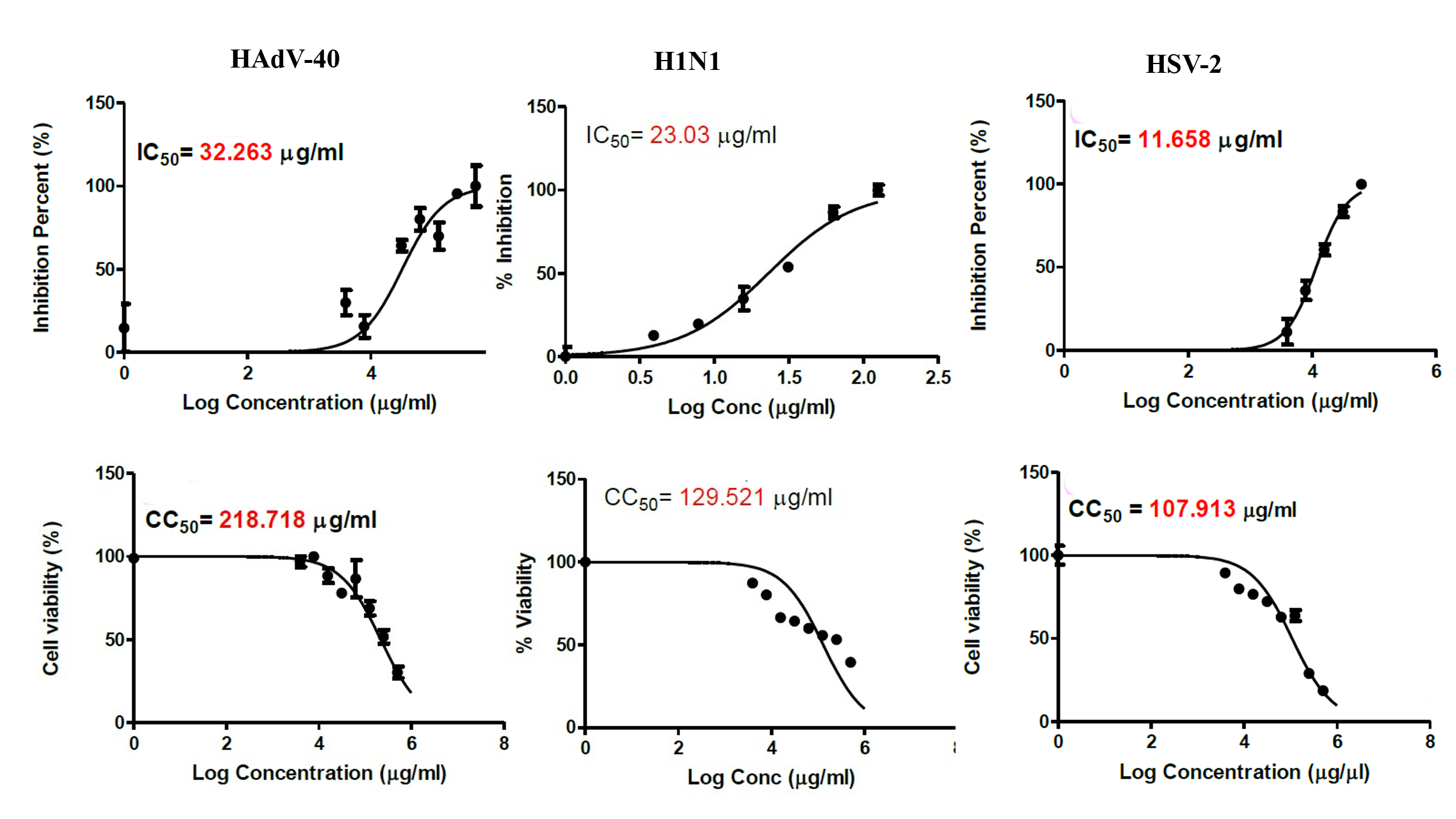


**Fig S6. Antiviral activities of the aerial flowering plant extracts of *R. patula*.**

**Table (S1)** Molecular interactions of ligands with amino acids of human adenovirus type 40 (PDB ID 4PIE)

| No | **Protein** | **Ligand** | **3D Structure** | Hydrophilic Interactions | | **Hydrophobic Contacts** | | **No. of**  **H-Bonds** | **No. of**  **Total Bonds** | **affinity**  **kcal mol-1** |
| --- | --- | --- | --- | --- | --- | --- | --- | --- | --- | --- |
|  |  |  |  | Residue (H- Bond) | Length | **Residue (Bond type)** | **Length** |  |  |  |
| 1 | **human Adenovirus type 40 (PDB: ID 4PIE)** | **Ellagic acid** | **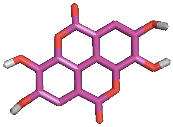** | Asn44, (H- Bond)  Asn44, (H- Bond)  Asn44, (H- Bond) | 2.54  2.53  2.71 | Trp55, (Pi-Pi-stacked)  Trp55, (Pi-Pi-stacked)  Gly52, (C-Hydrogen bond)  Gly52, (C-Hydrogen bond) | 5.21  6.79  3.73  3.25 | **3** | **7** | **-7.20** |
| 2 |  | **quercetin** | **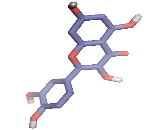** | Asn44, (H- Bond)  Glu5, (H- Bond) | 2.41  2.90 | Gly52, (C-Hydrogen bond)  Arg48, (Pi-alkyl)  Gly2, (Pi-Cation)  Trp55, (Pi-Pi-stacked)  Trp55, (Pi-Pi-stacked)  Ser4, (C-Hydrogen bond) | 3.39  5.48  4.79  4.95  6.91  3.53 | **2** | **8** | **-6.70** |
| 3 |  | **Rutin** | 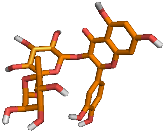 | Gly51, (H- Bond)  Gln6, (H- Bond)  Ser3, (H- Bond) | 2.90  1.90  2.52 | Arg48, (Pi-alkyl)  Gly51, (C-Hydrogen bond) | 4.15  3.24 | **3** | **5** | **-6.90** |
| 4 |  | **Naringenin** | 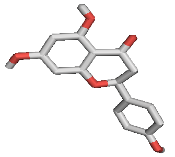 | Val53, (H- Bond)  Asn44, (H- Bond) | 1.87  5.29 | Gly51, (C-Hydrogen bond)  Arg48, (Pi-alkyl)  Trp55, (Pi-Pi-stacked)  Trp55, (Pi-Pi-stacked) | 2.89  4.68  5.31  4.72 | **2** | **6** | **-6.20** |
| 5 |  | **Hesperetin** | 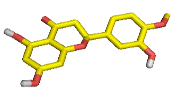 | Ser119, (H- Bond)  Gln115, (H- Bond) | 2.71  1.82 | Ala120, ((Pi-alkyl)  Cys122, ((Pi-alkyl)  Leu201, ((Pi-alkyl)  Val53, (C-Hydrogen bond) | 5.26  6.11  5.12  3.56 | **2** | **6** | **-6.90** |
| 6 |  | **Kaempferol** | 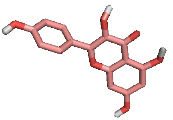 | Asn44, (H- Bond) | 2.87 | Gly52, (C-Hydrogen bond)  Arg48, (Pi-alkyl)  Gly2, (Pi-Cation)  Trp55, (Pi-Pi-stacked)  Trp55, (Pi-Pi-stacked)  Ser4, (C-Hydrogen bond)) | 3.36  5.47  4.85  6.88  4.95  3.51 | **1** | **8** | **-6.70** |
| 7 |  | **Gallic acid**  **(standard)** | 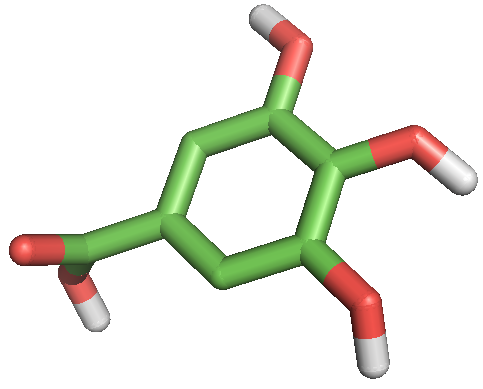 | Tyr25, (H- Bond)  Glu5, (H- Bond) | 2.65  2.87 | Arg48, (Pi-alkyl)  Trp55, (Pi-Pi-stacked) | 5.45  5.28 | **2** | **4** | **-5.00** |

**Table (S2)** Molecular interactions of ligands with amino acids of Herpes simplex virus type 2 proteases (PDB ID 1AT3):

|  | **Protein** | **Ligand** | **3D Structure** | **Hydrophilic Interactions** | | **Hydrophobic Contacts** | | **No. of**  **H-Bonds** | **No. of**  **Total Bonds** | **affinity**  **kcal mol-1** |
| --- | --- | --- | --- | --- | --- | --- | --- | --- | --- | --- |
|  |  |  |  | **Residue (H- Bond)** | **Length** | **Residue (Bond type)** | **Length** |  |  |  |
| 1 | **proteases of Herpes simplex virus type 2** | **Ellagic acid** | **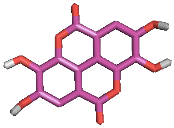** | **Leu130, (H- Bond)**  **Lys133, (H- Bond)**  **Arg157, (H- Bond)**  **Arg156, (H- Bond)**  **Ile154, (H- Bond)** | **3.06**  **2.50**  **2.15**  **5.32**  **2.50** | **His61, (Pi-Pi T shaped)**  **His61, (Pi-Pi T shaped)**  **His61, (Pi-Pi T shaped)**  **Leu38, (Pi-alkyl)**  **Arg156, (Pi-alkyl)**  **Arg156, (Pi-Cation)**  **Ser129, (Pi-Lone Pair)** | **4.92**  **5.36**  **4.91**  **5.34**  **4.42**  **3.51**  **2.87** | **5** | **15** | **-6.30** |
| 2 |  | **quercetin** | **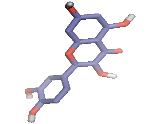** | **Thr132, (H- Bond)**  **Thr132, (H- Bond)**  **Lys133, (H- Bond)**  **Arg156, (H- Bond)**  **Arg156, (H- Bond)** | **2.20**  **2.50**  **2.38**  **3.41**  **2.60** | **Leu38, (Pi-alkyl)**  **Lys133, (Pi-Cation)**  **His61, (Pi-Cation)**  **His61, (Pi-Cation)** | **5.35**  **3.46**  **4.95**  **4.60** | **6** | **10** | **-6.30** |
| 3 |  | **Rutin** | 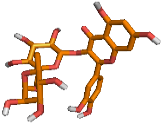 | **Ser131, (H- Bond)**  **Arg156, (H- Bond)**  **Arg157, (H- Bond)**  **Ser215, (H- Bond)** | **2.20**  **2.66**  **1.96**  **2.21** | **Leu38, (Pi-alkyl)**  **Arg62, (Pi-alkyl)**  **Asp60, (Pi-Anion)**  **Asp60, (Pi-Anion)** | **5.12**  **5.02**  **4.08**  **3.72** | **4** | **9** | **-7.90** |
| 4 |  | **Chlorogenic acid** | 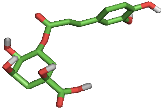 | **Arg157, (H- Bond)**  **Arg157, (H- Bond)**  **Ser129, (H- Bond)**  **Asp60, (H- Bond)**  **Asp60, (H- Bond)**  **Asn220, (H- Bond)**  **Asn220, (H- Bond)** | **2.51**  **2.85**  **2.80**  **2.76**  **2.07**  **2.14**  **2.22** | **His61, (Pi-Pi T shaped)**  **Arg156, (Pi-Cation)** | **5.67**  **4.39** | **7** | **10** | **-7.00** |
| 5 |  | **Hesperetin** | 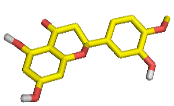 | **Ser129, (H- Bond)**  **Arg157, (H- Bond)** | **2.53**  **2.39** | **Arg156, (Pi-Cation)**  **His61, (Carbon H- Bond)**  **Thr132, (Carbon H- Bond)**  **Cys152, (Pi-alkyl)**  **Leu38, (Pi-alkyl)** | **4.50**  **3.64**  **3.41**  **5.09**  **5.47** | **2** | **7** | **-6.60** |
| 6 |  | **Catechin** | 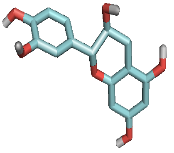 | **Thr132, (H- Bond)**  **Thr132, (H- Bond)**  **Lys133, (H- Bond)** | **2.45**  **2.68**  **2.83** | **Leu513, (Pi-alkyl)**  **Arg156, ((Pi-Pi stacked)**  **His61, (Pi-Cation)**  **Lys133, (Pi-Cation)** | **5.13**  **4.75**  **4.90**  **3.67** | **3** | **7** | **-6.40** |
| 7 |  | **Acyclovir**  **(standard)** | 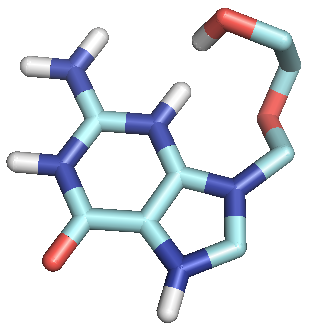 | **Asp60, (H- Bond)**  **Asp60, (H- Bond)**  **Asp60, (H- Bond)**  **Asn220, (H- Bond)**  **Arg156, (H- Bond)** | 2.16  2.46  2.33  2.25  2.66 | Cys152, (Pi-alkyl) | 4.11 | **5** | **6** | **-5.30** |

**Table (S3)** Molecular interactions of ligands with amino acids of neuraminidase of influenza virus (H1N1), (PDB: ID 3B7E):

|  | **Protein** | **Ligand** | **3D Structure** | **Hydrophilic Interactions** | | **Hydrophobic Contacts** | | **No. of**  **H-Bonds** | **No. of**  **Total Bonds** | **affinity**  **kcal mol-1** |
| --- | --- | --- | --- | --- | --- | --- | --- | --- | --- | --- |
|  |  |  |  | **Residue (H- Bond)** | **Length** | **Residue (Bond type)** | **Length** |  |  |  |
| 1 | **neuraminidase of influenza virus (H1N1)** | **Ellagic acid** | **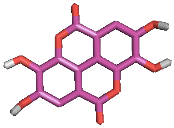** | Glu276, (H- Bond)  Glu276, (H- Bond)  Arg292, (H- Bond)  Asp151, (H- Bond)  Glu119, (H- Bond)  Trp178, (H- Bond) | 2.99  3.06  1.92  2.24  2.58 | Arg224, (Pi-alkyl)  Arg224, (Carbon-H)  Ser246, (Carbon-H)  Arg152, (Carbon-H)  Glu277, (Pi-Cation)  Glu227, (Pi-Anion) | 5.24  3.22  3.59  3.76  3.87  4.97 | **6** | **12** | **-8.20** |
| 2 |  | **quercetin** | **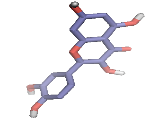** | Glu277, (H- Bond)  Arg224, (H- Bond)  Gly244, (H- Bond)  Asp151, (H- Bond)  Ser246, (H- Bond) | 1.73  2.56  2.72  2.33  2.90 | Ile222, (Pi-Alkyl)  Ile222, (Pi-Sigma)  Asp151, (Pi-Anion) | 4.23  4.14  1.82 | **5** | **8** | **-8.60** |
| 3 |  | **Rutin** | **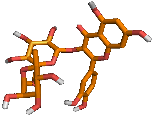** | Glu227, (H- Bond)  Glu119, (H- Bond)  Arg118, (H- Bond)  Arg118, (H- Bond)  Arg292, (H- Bond)  Asn347, (H- Bond) | 1.94  2.50  2.81  2.18  2.70  2.44 | Arg152, (Pi-Anion)  Ile222, (Pi-Alkyl)  Ile222, (Pi-Sigma)  Arg224, (Pi-Sigma)  Asp151, (Carbon-H) | 4.06  4.67  3.37  3.25  3.24 | **6** | **13** | **-8.90** |
| 4 |  | **Catechin** | 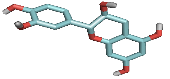 | Glu227, (H- Bond)  Asp151, (H- Bond)  Gly244, (H- Bond)  Asn221, (H- Bond)  Ser246, (H- Bond) | 2.43  2.39  2.75  2.95  2.84 | Ile222, (Pi-Sigma)  Asp151, (Pi-Anion) | 3.36  4.76 | **5** | **7** | **-8.20** |
| 5 |  | **Hesperetin** | 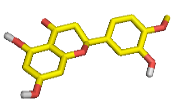 | Glu277, (H- Bond)  Asp151, (H- Bond)  Gly244, (H- Bond) | 2.37  2.21  2.29 | Ile222, (Pi-Sigma)  Asp151, ((Pi-Anion) | 3.29  4.91 | **3** | **5** | **-8.20** |
| 6 |  | **Kaempferol** | 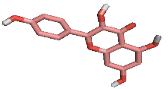 | Glu277, (H- Bond)  Asp151, (H- Bond)  Gly244, (H- Bond) | 1.93  1.98  2.55 | Ile222, (Pi-Sigma)  Asp151, ((Pi-Anion)  Ile222, (Pi-Alkyl) | 3.41  4.84  5.38 | **3** | **6** | **-8.10** |
| 7 |  | **Oseltamivir**  **(standard)** | 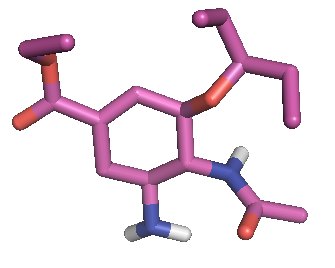 | Arg292, (H- Bond)  Arg118, (H- Bond)  Arg371, (H- Bond)  Glu119, (H- Bond)  Arg152, (H- Bond) | 2.01  2.33  1.99  2.31  2.05 | Ile222, (Pi-Alkyl)  Glu277, (Carbon-H) | 4.17  3.74 | **5** | **2** | **-6.30** |
